# Drug Delivery Systems for Resiquimod to Control Myeloid‐Derived Suppressor Cells in Cancer Immunotherapy

**DOI:** 10.1002/wnan.70052

**Published:** 2026-03-09

**Authors:** Yanying He, Yoon Yeo

**Affiliations:** ^1^ Department of Industrial and Molecular Pharmaceutics Purdue University West Lafayette Indiana USA; ^2^ Weldon School of Biomedical Engineering, Purdue University West Lafayette Indiana USA; ^3^ Purdue University Institute for Cancer Research West Lafayette Indiana USA

**Keywords:** cancer immunotherapy, drug delivery, immunosuppression, myeloid‐derived immunosuppressor cells, resiquimod, TLR7 agonist

## Abstract

Over the past decade, immunotherapy has emerged as the fourth pillar of cancer therapy, following surgery, chemotherapy, and radiotherapy. However, tumors often evade immune responses by altering the tumor microenvironment (TME), which recruits immunosuppressive cells such as myeloid‐derived suppressor cells (MDSCs), tumor‐associated macrophages, and regulatory T cells. Among these, MDSCs are considered key contributors to immunotherapy failure and have become major targets in new therapeutic strategies. Growing evidence indicates that controlling MDSCs is critical to the success of cancer immunotherapy, and several drug classes have shown feasibility. In this review, we introduce the significance of MDSCs as a target in cancer immunotherapy and highlight different therapeutic approaches to counteract their immunosuppressive functions. We discuss recent efforts to optimize drug delivery for controlling MDSCs, focusing on resiquimod (R848) as a representative drug candidate.

## Graphical abstract

Myeloid‐derived suppressor cells (MDSCs) are key contributors to cancer immunotherapy failure and major targets in new therapeutic strategies. We highlight therapeutic approaches to counteract their immunosuppressive functions and discuss recent efforts to optimize drug delivery for controlling MDSCs, focusing on resiquimod as a representative drug candidate.

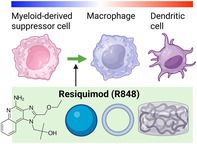

## 1 Introduction

Cancer remains one of the most significant public health challenges worldwide. Approximately 20 million cases are diagnosed, and nearly 10 million cancer‐related deaths occur worldwide every year (Bray et al. 2024). Notably, lung cancer continues to be the leading cause of cancer death, with an estimated 1.8 million deaths, accounting for one‐fifth of the cancer deaths, followed closely by colorectal, liver, breast, and stomach cancers (Bray et al. 2024). Cancer control has relied on three therapeutic pillars in the previous 60 years: surgery, chemotherapy, and radiotherapy (Yuzhalin 2024). These modalities, as well as continuously developing combinational therapies, have improved patient survival.

Meanwhile, immunotherapy has emerged over the past decade as the fourth pillar of cancer immunotherapy. Immunotherapy leverages the body's innate and adaptive immune system to combat cancer cells, based on immune‐activating cytokines or small molecules, immune‐checkpoint blockade (ICB), adoptive cell transfer, and therapeutic cancer vaccines (Hunter 2017). Various immune checkpoint inhibitors, preventing PD‐1/PD‐L1 or CTLA‐4 from inactivating cytotoxic T cells, have been approved for clinical use in the 2010s for treating dozens of tumor types (Lee et al. 2022). Adoptive transfer of chimeric antigen receptor T (CAR‐T) cells was approved for the treatment of hematologic malignancies (Ikeda 2025). CAR‐T cells are engineered to target tumor surface antigen, such as CD19 or B‐cell maturation antigen (BCMA). In 2024, the US Food and Drug Administration approved the first tumor‐infiltrating lymphocyte (TIL) therapy, Lifileucel (Amtagvi), autologous tumor‐specific T cells. TILs are extracted from a patient, expanded ex vivo, and reinfused to the patient, for the treatment of PD‐1‐refractory metastatic melanoma (Kaur et al. 2025). Currently, multiple in vivo CAR‐T therapies aiming to generate CAR‐T cells directly within the patient are tested in non‐human primates and phase I clinical trials (Bot et al. 2025; Hunter et al. 2025). Additionally, therapeutic cancer vaccines, such as talimogene laherparepvec (T‐VEC) and Sipuleucel‐T, were approved for melanoma and prostate cancer, respectively. T‐VEC is an intratumorally injected, granulocyte‐macrophage colony‐stimulating factor (GM‐CSF)‐encoding oncolytic virus, derived from human herpes simplex virus 1. T‐VEC recruits and activates antigen‐presenting cells (APCs) to the injection site by killing tumor cells and expressing GM‐CSF (Pol et al. 2016). Sipuleucel‐T is an autologous dendritic cell vaccine, which is expanded ex vivo and pulsed with PA2024, a recombinant fusion protein consisting of prostate cancer antigen and GM‐CSF. The antigen‐fed dendritic cells are reinfused to prime and activate T cells in the patients (Anassi and Ndefo 2011; Harzstark and Small 2008).

However, tumor cells can evade these treatments by dampening the immune surveillance and rendering the current immunotherapy ineffective (Ibrahim et al. 2025). The tumor microenvironment (TME) significantly affects the effectiveness of immunotherapy, often through the recruitment and activation of immunosuppressive cells, such as myeloid‐derived suppressor cells (MDSCs), tumor‐associated macrophages (TAMs), and regulatory T (Treg) cells (Zhao et al. 2023). Due to the prevalence in TME and the ability to suppress other immune cells and expand immunosuppressive cells, MDSCs are considered the main contributors to the failure of cancer immunotherapy and have emerged as key targets in recent therapeutic strategies (Ibrahim et al. 2025; Wang, Song, et al. 2018; Zhao et al. 2023).

In this review, we introduce the significance of MDSCs as a target in cancer immunotherapy and different therapeutic approaches to counteract their immunosuppressive functions. We discuss recent efforts to optimize drug delivery for controlling MDSCs, using resiquimod as a representative drug candidate.

## 2 MDSC as a Target in Cancer Immunotherapy

### 2.1 Significance of MDSCs in Cancer

The prevalence of MDSCs correlates with poor prognosis across multiple cancer types. Circulating MDSCs increased in breast cancer patients compared to healthy individuals and showed a positive correlation with the cancer progression stage and metastasis (Bergenfelz et al. 2015; Diaz‐Montero et al. 2009). Similarly, the frequency of circulating and intratumoral MDSCs increased in colorectal carcinoma patients, showing a positive correlation with the tumor stage and metastasis (Sun et al. 2012). In a mouse model of CT26 colon cancer, tumors grew faster when co‐inoculated with MDSCs than with CT26 cells alone (Spinetti et al. 2016). MDSCs also pose a significant barrier to the efficacy of current treatments, such as chemotherapy and immune checkpoint inhibitors. The increased MDSC frequency in cancer patients is associated with treatment failure in various cancers, including ovarian cancer (Santegoets et al. 2018), lung cancer (Bronte et al. 2022), and melanoma (Petrova et al. 2023). A meta‐analysis showed that the MDSC population had a generally negative correlation with the survival rate in cancer patients receiving clinical treatments, with breast cancers showing the most adverse correlation among various tumor types (Wang, Song, et al. 2018).

Cancer therapies can further increase the number of MDSCs. Many cancer patients showed elevated MDSC frequency after receiving radiation therapy (Bergerud et al. 2024). MDSC levels were elevated in cancer patients receiving doxorubicin (DOX)‐cyclophosphamide combination (Diaz‐Montero et al. 2009) or paclitaxel (Wesolowski et al. 2017). Many chemotherapeutic agents induce immunogenic cell death, causing the release of high mobility group B1 (HMGB1), a damage‐associated molecular pattern (DAMP) (Kwon et al. 2024), which can promote MDSC generation (Ostrand‐Rosenberg and Fenselau 2018). Immunotherapy also increases MDSCs. The MDSC level was elevated in prostate cancer patients receiving a personalized peptide vaccine (Koga et al. 2017). ICB therapies, such as anti‐PD‐1 antibody treatment, significantly increased the intratumoral MDSC level in melanoma patients (Sun et al. 2021; Theivanthiran et al. 2020), likely via PD‐L1/NLRP3 inflammasome activation (Theivanthiran et al. 2020). These studies indicate that MDSCs mediate negative feedback to cancer therapies and the evolution of treatment resistance.

Consistent with the negative implication of MDSCs, preclinical studies and emerging clinical evidence have shown that targeting MDSCs can enhance therapeutic effects of existing cancer treatments (Armstrong et al. 2024; Bosiljcic et al. 2019; Tobin et al. 2023; Turco et al. 2023; Zhang, Jin, et al. 2023). Accordingly, various therapeutic strategies have been developed to downregulate or modulate MDSCs, thereby reducing resistance to conventional therapies and restoring their antitumor activities.

### 2.2 MDSC Identification

MDSCs refer to a heterogeneous population of immature myeloid cells. In mice, MDSCs are typically defined as cells that co‐express specific surface markers, CD11b^+^ and Gr‐1^+^ (epitopes common to Ly6C and Ly6G antigens) (Bronte et al. 2016). MDSCs can be further divided into two main subtypes based on their morphology and function: monocytic MDSCs (M‐MDSCs: CD11b^+^ Ly6G^−^ Ly6C^hi^) and polymorphonuclear or granulocytic MDSCs (PMN‐ or G‐MDSCs: CD11b^+^ Ly6G^+^ Ly6C^lo^) (Bronte et al. 2016). The latter is also a marker of neutrophils in mice, which are classified into two distinct types: N1 neutrophils, with antitumor activity, and N2 neutrophils, also known as tumor‐associated neutrophils (TANs), that promote tumor angiogenesis and suppress T cell activity (Fridlender et al. 2009). PMN‐MDSCs and N2 neutrophils are similar in morphology, phenotype, and function (Bronte et al. 2016). While they may be distinguished by the maturation status (Sagiv et al. 2015) or single cell transcriptome analysis (Veglia et al. 2021), PMN‐MDSCs are largely considered synonymous with N2 neutrophils due to their phenotypic and functional similarities (Akkari et al. 2024; Groth et al. 2019; Marvel and Gabrilovich 2015; Veglia et al. 2021).

In humans, the Gr‐1 epitope is absent; thus, MDSCs are classified as M‐MDSC (CD33^+^ CD11b^+^ CD14^+^ CD15^−^ HLA‐DR^lo^/^−^), PMN‐MDSC (CD33^+^ CD11b^+^ CD14^−^ CD15^+^ CD33^+^ or CD11b^+^ CD14^−^ CD15^+^ CD66b^+^ CD16^+^ HLA‐DR^lo^/^−^), and early‐stage immature MDSC (lineage‐negative, HLA‐DR^−^, CD33^+^) (Akkari et al. 2024; Bronte et al. 2016).

### 2.3 Origin and Development of MDSCs

**FIGURE 1:**
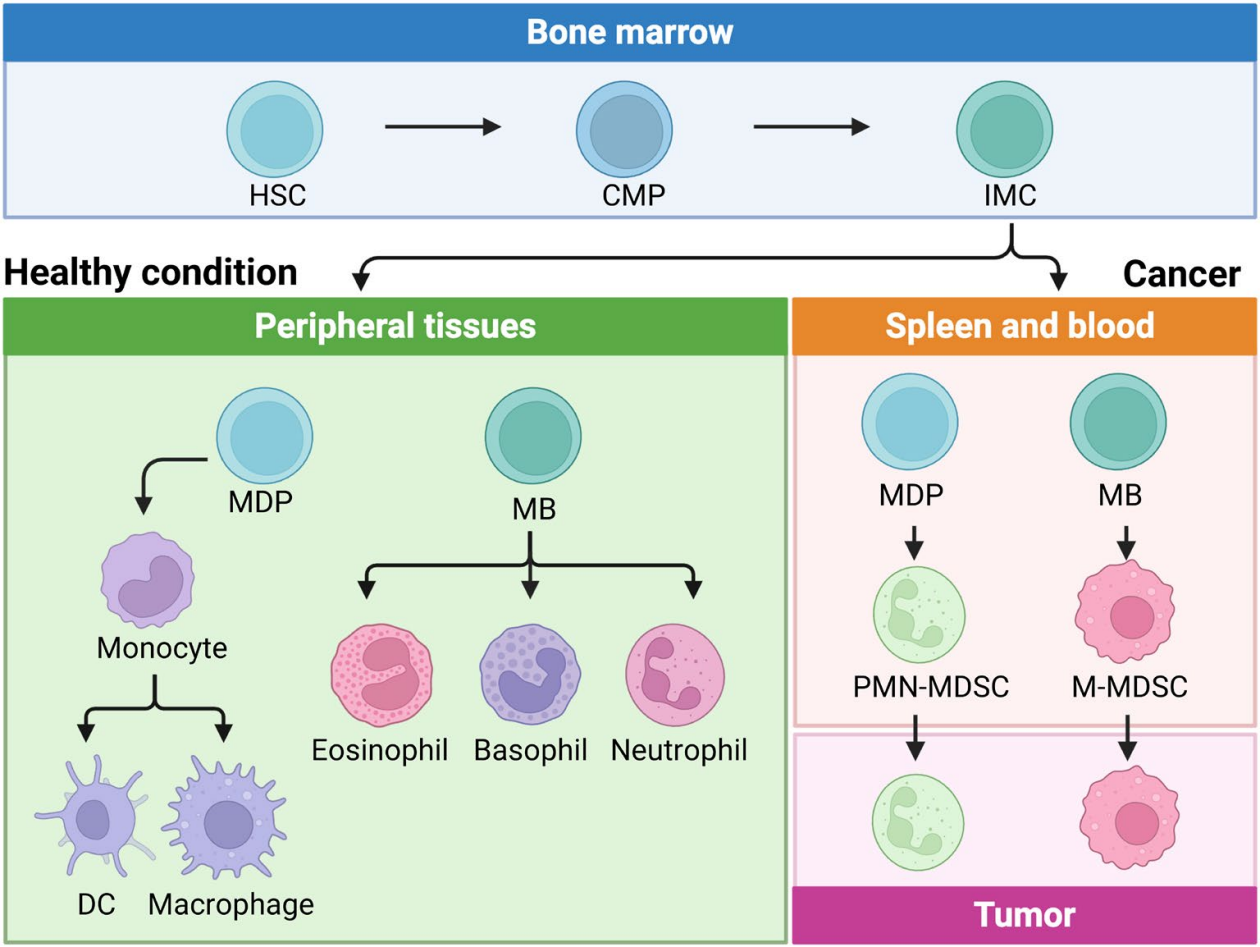
Schematic of MDSCs development. In the bone marrow, hematopoietic stem cells (HSCs) differentiate to common myeloid progenitors (CMPs), which expand into immature myeloid cells (IMCs). IMCs become macrophage/dendritic cell progenitors (MDPs) or myeloblasts (MBs). In normal physiological conditions, MDPs further differentiate into macrophages and dendritic cells (DCs), whereas MBs differentiate into eosinophils, basophils, and neutrophils. Under cancer conditions, IMCs are differentiated into M‐MDSCs and PMN‐MDSCs in the spleen and blood, which are recruited to the tumor. Adapted from Li, Shi, et al. (2021). Created with Biorender.com.

MDSCs are generated primarily in the bone marrow from common myeloid progenitor cells (Groth et al. 2019). Under normal physiological conditions, these progenitor cells differentiate into mature myeloid cells, such as dendritic cells (DCs), monocytes, and granulocytes (Gabrilovich et al. 2012). However, in the context of cancer, various tumor‐associated factors and the inflamed TME disrupt this differentiation process, letting immature myeloid cells expand and become MDSCs (Figure 1). Granulocyte macrophage colony‐stimulating factor (GM‐CSF) (Serafini et al. 2004), IL‐6 (Walker et al. 2008), IL‐1*β*, Bv8 (Tannenbaum et al. 2019), tumor necrosis factor *α* (TNF‐*α*) (Atretkhany et al. 2016), and 5′‐AMP (Chiu et al. 2017) cause MDSC expansion. Upon binding to these factors, signal transducer and activator of transcription 3 (STAT3) is activated, which stimulates myelopoiesis and prevents further maturation of the MDSCs (Gabrilovich and Nagaraj 2009). Moreover, spleen undergoes a similar process as bone marrow, called extramedullary myelopoiesis, providing additional source of MDSCs (Cortez‐Retamozo et al. 2012).

Once MDSCs are generated, they are recruited to the tumor site via a complex interaction of tumor‐secreted chemokines and cytokines or by other components within the TME. Common chemokines that facilitate this recruitment include CCL2, CCL5, and CXCL5, which bind to their receptors on MDSCs, such as CXCR1 and CXCR2, thereby promoting their migration (Lazennec et al. 2024; Oo et al. 2022; Tannenbaum et al. 2019). Additionally, various tumor‐expressed factors, including indoleamine 2,3‐dioxygenase (IDO), colony‐stimulating factor‐1 (CSF‐1), hypoxia‐inducible factor‐1 (HIF‐1), and vascular endothelial growth factor (VEGF) recruit MDSCs to the tumor (Chiu et al. 2017; Holmgaard et al. 2016, 2015; Horikawa et al. 2017).

### 2.4 Mechanisms of MDSC‐Mediated Immunosuppression

MDSCs play a critical role in developing an immunosuppressive TME that fosters tumor growth and progression. MDSCs can suppress various immune cells, including T cells, natural killer (NK) cells, and DCs, and the mechanism of suppression also varies depending on the MDSC subtype (Gabrilovich and Nagaraj 2009).

**FIGURE 2:**
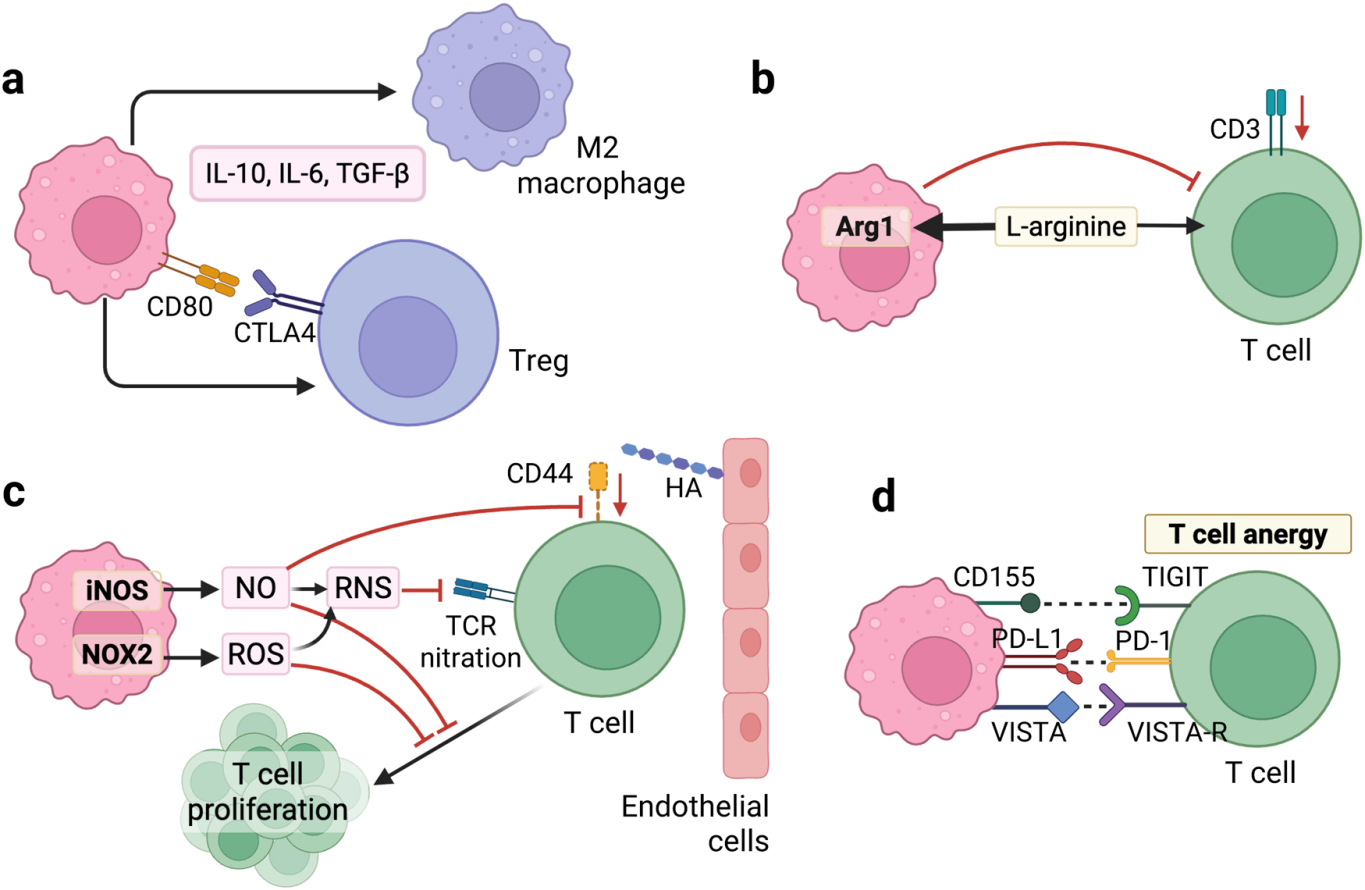
Mechanisms of MDSC‐mediated immunosuppression. (a) MDSCs recruit M2 macrophages and Treg. (b) MDSCs deplete L‐arginine and downregulate CD3 receptors on T cells to prevent T cell activation. (c) MDSCs produce nitric oxide (NO) and reactive oxygen species (ROS), both of which inhibit T cell proliferation. NO also downregulates CD44 and prevents T cell homing. NO reacts with ROS to produce reactive nitrogen species (RNS), which nitrate the T cell receptors (TCR). (d) MDSCs express immune checkpoints and induce T cell anergy. Adapted from Groth et al. (2019). Created with Biorender.com.

Immunosuppressive cytokines produced by MDSCs, such as interleukin‐10 (IL‐10), transforming growth factor‐beta (TGF‐*β*), and IL‐6, suppress the function and proliferation of innate and adaptive immune cells and activate M2 anti‐inflammatory macrophages and regulatory T cells (Treg) in tumor (Figure 2a) (Koh et al. 2020; Park et al. 2018). For instance, IL‐10 produced by MDSCs has been shown to correlate with poor overall survival in cancer patients (Koh et al. 2020).

Arginase‐1 (Arg1) and inducible nitric oxide synthase (iNOS) in the MDSCs mediate immunosuppression by depleting an essential amino acid, L‐arginine. L‐arginine is critical for T cell function and proliferation. Depletion of L‐arginine decreased the expression of CD3*ζ* in T cells, subsequently reducing their activation (Figure 2b) (Bronte et al. 2003; Zea et al. 2005). Both MDSC subtypes express Arg1, but iNOS is predominantly expressed in M‐MDSCs (Gabrilovich and Nagaraj 2009). iNOS uses L‐arginine to produce nitric oxide (NO), which downregulates the CD44 receptor on T cells, leading to impaired T cell extravasation to the tumor site (Figure 2c) (Schouppe et al. 2013). NO also negatively regulates the proliferation of T cells and the function of NK cells (Mazzoni et al. 2002; Stiff et al. 2018).

Reactive Oxygen Species (ROS), such as superoxide (O_2_^−^) and hydroxyl radicals, along with non‐radical molecules such as hydrogen peroxide (H_2_O_2_), are upregulated in MDSCs and contribute to the inhibition of T cell activation (Figure 2c) (Li, Shi, et al. 2021). Increased expression of NADPH oxidase (NOX2) in the MDSCs is responsible for the high ROS level in MDSCs (Corzo et al. 2009). While the elevated ROS level can cause cytotoxicity in normal cells, MDSCs thrive in this condition due to the presence of NF erythroid 2‐related factor 2 (Nrf2), a transcription factor that regulates oxidative stress (Beury et al. 2016). ROS can react with NO and produce peroxynitrite, which nitrates the T cell receptors on CD8 T cells and impairs their interaction with major histocompatibility complex (MHC) (Figure 2c) (Nagaraj et al. 2007).

Tregs are attracted to the tumor site by MDSCs, where their immunosuppressive functions further develop. MDSCs attract Tregs to the tumor site by secreting chemokines, including various C‐C chemokine receptor type 5 (CCR5) ligands, that bind to the highly expressed CCR5 on the Tregs (Schlecker et al. 2012). Their interaction can also be facilitated by the binding of CD80 on the MDSCs and the cytotoxic T‐lymphocyte‐associated protein 4 (CTLA4) on Tregs (Figure 2a) (Yang et al. 2006). Targeting CD80 by antibody or small interference RNA (siRNA) alleviated Treg‐mediated immunosuppression (Yang et al. 2006). In addition, MDSCs can induce de novo generation of Tregs by producing IL‐10 and TGF‐*β* (Huang et al. 2006).

MDSCs can suppress the function of immune cells via the immune checkpoint pathway (Figure 2d). PD‐L1 expression on the MDSCs in the tumor is significantly elevated due to the hypoxic environment, compared to those in the other organs, such as the spleen (Noman et al. 2014). Upon binding to the PD‐1 on the T cells, MDSCs induce T cell anergy (Noman et al. 2014). CTLA‐4 expression on MDSCs is also elevated and boosts arginase‐1 transcription (Liu et al. 2009). Moreover, MDSCs express a monocyte‐specific checkpoint, V‐domain Ig suppressor of T‐cell activation (VISTA), which is highly expressed in leukemia patients (Wang, Jia, et al. 2018). In the acidic environment created by hypoxia, VISTA binds to T cells and induces immunosuppression (Deng et al. 2024). M‐MDSCs secrete galectin‐9 that binds to TIM‐3 on CD8^+^ cells, which has been linked to the resistance to anti‐PD‐1 therapy (Limagne et al. 2019). PMN‐MDSCs interact with T cells via CD155, which binds to the T cell immunoreceptor with Ig and ITIM domains (TIGIT) and mediates T cell suppression (Monteran et al. 2024).

It is worth noting that the immunosuppressive activities of MDSCs vary between the spleen and the tumor in the potency, mechanism, and antigen specificity of the immunosuppression. The difference comes from the composition of MDSCs (M‐MDSCs vs. PMN‐MDSCs) and the extent of immune checkpoint receptor expression (Kumar et al. 2016).

## 3 Therapeutic Strategies to Target MDSCs

**FIGURE 3:**
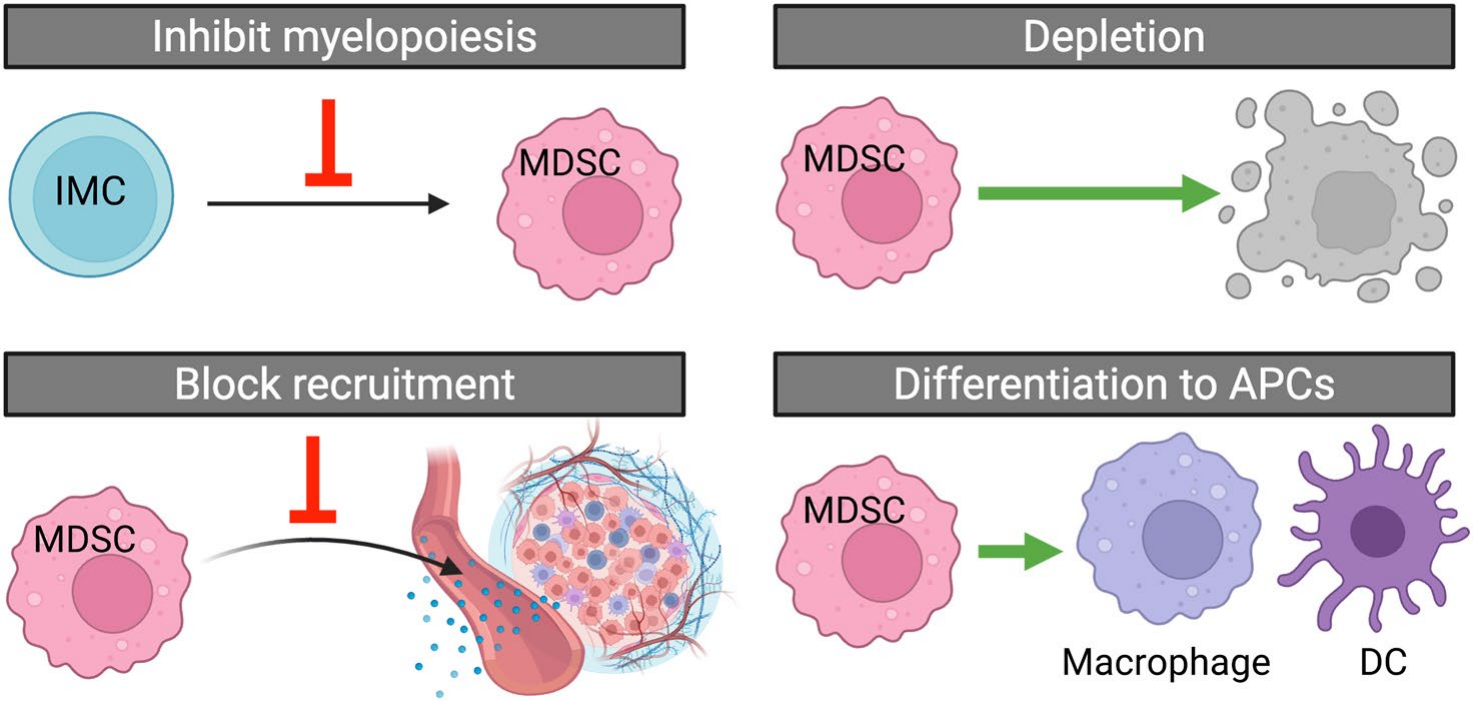
Strategies to control MDSCs. Inhibition of myelopoiesis to prevent MDSC generation. Blocking the recruitment of MDSCs to the TME. Direct depletion of MDSCs. Differentiation of MDSCs to macrophage and DCs. Created with Biorender.com.

MDSCs orchestrate immune paralysis, fuel tumor growth, and limit the efficacy of many anti‐cancer therapies. Therefore, cancer immunotherapy has focused on dismantling MDSC‐mediated immunosuppressive barriers. The efforts to control MDSCs include inhibiting myelopoiesis, preventing MDSC recruitment, depleting MDSCs, blocking MDSC functions, and promoting MDSC differentiation (Figure 3).

### 3.1 Inhibiting Myelopoiesis

Tumors hijack bone marrow and splenic myelopoiesis by secreting various tumor‐associated factors, which activate the STAT3 transcription factor and drive the continuous production of immature, immunosuppressive myeloid cells. Cutting this supply line of myelopoiesis helps control the MDSC population. The colony‐stimulating factor‐1 receptor (CSF‐1R) on MDSCs binds to colony‐stimulating factor‐1 (CSF‐1), resulting in the proliferation and differentiation of myeloid cells (Xu et al. 2013). Small molecule CSF‐1R inhibitor pexidartinib (PLX3397) decreased the intratumoral and splenic levels of MDSCs induced by radiation therapy (Xu et al. 2013). Cabiralizumab, a monoclonal antibody to CSF‐1R, was combined with PD‐1 blockade in a phase II clinical trial for T cell lymphoma patients (NCT 03927105). Tumor‐derived GM‐CSF promotes MDSC generation from their precursor and induces MDSC proliferation (Bayne et al. 2012; Kumar et al. 2022). Anti‐GM‐CSF antibody inhibited the precursor differentiation to MDSCs (Gargett et al. 2016), thereby reducing the intratumoral MDSC population (Bayne et al. 2012).

Myelopoiesis‐stimulating cytokines can also be targeted to suppress MDSC generation. IL‐6 is a key regulator of MDSC proliferation (Walker et al. 2008). Inhibition with anti‐IL‐6 receptor antibody lowered the MDSC population in the spleen and tumor and controlled the tumor growth (Sumida et al. 2012). Anti‐IL‐6 antibody also controlled the intratumoral MDSC level when combined with anti‐CTLA‐4 antibody and improved the antitumor efficacy (Hailemichael et al. 2022). However, in a transgenic melanoma model that overexpresses IL‐6, IL‐6 blockade therapy did not decrease MDSC frequency but enhanced tumor progression, suggesting complex roles of IL‐6 (Weber et al. 2020). IL‐6 can also activate CD8 T cells as a pro‐inflammatory cytokine; therefore, IL‐6 inhibition might have brought a conflicting consequence to the overall antitumor effect.

STAT3 is the downstream transcription factor that regulates myelopoiesis and prevents the maturation of myeloid cells (Gabrilovich and Nagaraj 2009). STAT3 inhibition is another strategy to control MDSCs without affecting the antitumor immune response of other immune cells. A phase I clinical trial using an FDA‐approved STAT3 inhibitor, ruxolitinib, in combination with anti‐PD‐1 antibody showed a drastic decrease in the MDSC population and a 50% response rate in patients with Hodgkin lymphoma (Zak et al. 2024). The combination of ruxolitinib with anti‐PD‐1 antibody was also tested in pre‐clinical pancreatic cancer models. The combinational therapy showed an improved antitumor efficacy, but mechanistic studies revealed that the efficacy was T‐cell‐dependent, with no significant change in MDSC frequency (Ajina et al. 2021; Lu et al. 2017). These results suggest MDSC inhibition by STAT3 blocking may vary with tumor type. BBI608, another STAT3 inhibitor, prevented MDSC generation, controlled MDSC suppression on T cells, and induced MDSC apoptosis (Bitsch et al. 2022). When combined with CAR‐T, BBI608 inhibited the function of MDSCs and enhanced the cytotoxic activity of CAR‐T in vitro (Guha et al. 2019). In addition, AZD9150, an antisense oligonucleotide inhibitor that downregulates STAT3 mRNA, showed a decrease in PMN‐MDSCs in three out of four patients with lymphoma (Reilley et al. 2018).

Histone deacetylase (HDAC) inhibitors prevent protein deacetylation (Cui et al. 2021). HDAC inhibitors, such as entinostat, inhibit the function of STAT3, downregulate Arg1 and iNOS, two of the main suppressive features of MDSCs, and decrease the expression of CCR2, which regulates MDSC recruitment (Cui et al. 2021). A phase II clinical study on entinostat, combined with an aromatase inhibitor, reduced the circulating MDSCs in breast cancer patients (Hellmann et al. 2021). However, entinostat combined with anti‐PD‐1 showed limited clinical efficacy on non‐small cell lung cancer, triple‐negative breast cancer, and advanced epithelial ovarian cancer (Hellmann et al. 2021).

### 3.2 Blocking MDSC Recruitment

MDSCs rely on chemokine receptors CXCR1 and CXCR2 for migration to tumors (Lazennec et al. 2024). Therapies targeting the chemotactic signaling can effectively reduce tumor‐associated MDSC levels. An antibody blocking CXCR2 significantly reduced the number of circulating and intratumoral MDSCs, thereby enhancing the efficacy of anti‐PD‐1 therapy in a preclinical rhabdomyosarcoma model (Highfill et al. 2014).

Cancer‐associated fibroblasts (CAFs) recruit MDSCs to the TME through the CXCR4 axis (Deng et al. 2017). CXCR4 blockade with a small molecule antagonist AMD3100 reduced the MDSC level in the blood and tumor, thereby decreasing the liver metastasis frequency in a preclinical colon tumor model (Benedicto et al. 2018). BL‐8040 (motixafortide) is a synthetic peptide that binds to CXCR4 with greater affinity and induces a more durable response than AMD3100 (Bockorny et al. 2020). Combination of BL‐8040 and anti‐PD‐1 showed a better response than standard therapy and decreased the intratumoral PMN‐MDSC level in the phase IIa clinical trial (Bockorny et al. 2020).

CCR2 and CCR5 are chemokine receptors expressed on MDSCs and interact with cognate ligands (CCLs) in tumor to infiltrate TME (Wang et al. 2022). CCR2 and CCR5 dual‐antagonist BMS‐687681 reduced the M‐MDSC population in the tumor treated with anti‐PD‐1 and radiotherapy, extending the survival time in mouse models of pancreatic ductal adenocarcinoma (Wang et al. 2022).

IL‐8, which is overexpressed in various tumors and further induced by chemotherapy, recruits MDSCs to the tumor site (Dominguez et al. 2017). Inhibition with a human anti‐IL‐8 antibody, HuMAX‐IL8, controlled the intratumoral PMN‐MDSC level in a preclinical study (Dominguez et al. 2017). In the phase I clinical trial, HuMAX‐IL8 achieved stable disease in the majority of the patients, although it did not change the MDSC level in peripheral blood (Bilusic et al. 2019).

CD11b is an adhesion molecule that binds to endothelial intercellular adhesion molecule–1 (ICAM‐1) and facilitates the extravasation of immune cells (Panni et al. 2019). A small‐molecule drug ADH‐503 activates the CD11b binding and strengthens the adhesion to the endothelial layer, thereby preventing the extravasation of immune cells (Maiguel et al. 2011). In the orthotopic pancreatic cancer models, ADH‐503 decreased the MDSC level in the tumor and improved the survival rate when combined with chemotherapy, immunotherapy, or ICB (Panni et al. 2019). It was tested in a phase I/II clinical study as monotherapy or in combination with anti‐PD‐L1 in patients with solid tumors (DeNardo et al. 2021). While preliminary results indicated a decrease in the peripheral MDSCs in the patients (Park et al. 2021), the clinical trial was terminated due to limited therapeutic efficacy (NCT04060342).

### 3.3 Depleting MDSCs

Another strategy to offset immunosuppressive effect of MDSCs is to deplete the MDSCs already present in the tumor. Some chemotherapeutics used to inhibit tumor cell proliferation can also deplete MDSCs. 5‐fluorouracil (5FU), gemcitabine, and docetaxel were shown to decrease the MDSC level in preclinical models (Bosiljcic et al. 2019; Kodumudi et al. 2010; Vincent et al. 2010). This effect was also observed in patients with pancreatic cancer induced by gemcitabine (Eriksson et al. 2016) and in patients with colon tumors treated with 5FU (Yang et al. 2023). However, unless myelopoiesis is controlled, newly generated MDSCs may continue to infiltrate the TME without continuous supply of the depleting agents. Therefore, MDSC‐depleting agents were combined with anti‐myelopoiesis therapy to improve therapeutic efficacy. Combination of anti‐IL‐6 receptor antibody and gemcitabine downregulated MDSCs and restored T cell responses in preclinical models (Sumida et al. 2012). Combination of a 5FU prodrug (capecitabine) and anti‐VEGF antibody, an antiangiogenic agent that can potentially block MDSC recruitment, reduced the circulating MDSC frequency in patients with glioblastoma (Peereboom et al. 2019).

Tyrosine kinase inhibitors (TKIs) block the function of various receptors, including VEGF receptor and stem cell factor receptor (ckit), to deplete MDSCs (Ozao‐Choy et al. 2009). Sunitinib, one of the approved TKIs for renal cell carcinoma, decreased the MDSC level in patients with renal cell carcinoma by inducing their apoptosis (Ko et al. 2009). In a phase II clinical trial, tivozanib lowered the MDSC frequency in the blood of patients with hepatocellular carcinoma (Kalathil et al. 2020). Other TKIs, such as ibrutinib and cabozantinib, also decreased the MDSC level in mouse models (Kwilas et al. 2014; Stiff et al. 2016).

Since MDSCs have specific surface markers, such as CD11b and Gr1 in mice and CD11b and CD33 in humans, antibodies that target these markers have been used to induce antibody‐dependent cellular cytotoxicity to MDSCs. An anti‐Gr1 antibody decreased the intratumoral MDSC frequency and increased T cell infiltration, thereby slowing tumor growth in a mouse ovarian cancer model (Horikawa et al. 2017). The anti‐Gr1 antibody was also combined with a TLR3 agonist, an MDSC‐polarizing agent, to enhance the antitumor efficacy of CAR‐T therapy (Di et al. 2019). However, the translation of anti‐Gr1 antibody is limited due to its transient effect, potential toxicity, and, importantly, the lack of Gr1 in humans (Ma and Greten 2016; Morales et al. 2009). CD11b is a shared epitope in mice and humans and has been targeted with antibodies (Siebert et al. 2020). However, the use of anti‐CD11b antibodies is limited by the dose‐dependent toxicities (DeNardo et al. 2021).

Translational efforts target the human MDSC marker CD33, which is expressed on both M‐MDSCs and PMN‐MDSCs. The anti‐CD33 antibody depleted patient‐derived MDSCs ex vivo and blocked their immunosuppressive functions (Eksioglu et al. 2017). However, anti‐CD33 antibody alone showed limited clinical efficacy in leukemia (Laszlo et al. 2014). Various drug conjugates to anti‐CD33 antibodies were developed to leverage the efficacy. An anti‐CD33 antibody‐calicheamicin (cytotoxic agent) conjugate, called gemtuzumab‐ozogamicin (GO or Mylotarg), was approved for the treatment of acute myeloid leukemia (Jen et al. 2018). When tested on patient‐derived MDSCs, GO decreased the MDSC level and restored the proliferation and functions of cytotoxic T cells or CAR‐T cells, suggesting its potential utility in CAR‐T therapy (Fultang et al. 2019). In addition, a bispecific CD33 and CD3 T cell engager, which depletes MDSCs and augments the proliferation and cytotoxicity of T cells, is undergoing clinical trials in various tumors (Cheng, Chen, et al. 2022).

### 3.4 Promoting MDSC Differentiation

An alternative to depleting MDSCs or blocking their activity is to promote the differentiation of MDSCs, leveraging their increased population in the TME, into antitumor myeloid cells, such as macrophages and DCs. Vitamin A derivatives, dihydroorotate dehydrogenase (DHODH) inhibitors, and toll‐like receptor (TLR) agonists have been explored for this purpose.

*All‐trans*‐retinoic acid (ATRA) is a vitamin A derivative that modulates the nuclear retinoic receptors, which, upon activation, increases the glutathione level and decreases ROS production, resulting in MDSC differentiation (Nefedova et al. 2007). ATRA stimulated the differentiation of patient‐derived MDSCs into antigen‐presenting cells (APCs) and upregulated the expression of MHC II and co‐stimulating factors, alleviating T cell suppression (Kusmartsev et al. 2008). ATRA, combined with IL‐2, induced a dose‐dependent decrease in MDSC levels in renal cell carcinoma patients (Mirza et al. 2006). ATRA was also combined with ICB, such as anti‐CTLA‐4 and anti‐PD‐1, in phase I/II clinical trials for the treatment of melanoma (Tobin et al. 2023, 2018). ATRA combined with anti‐PD‐1 was effective in controlling tumor progression and achieved complete response in 50% of the patients (Tobin et al. 2023). In responding patients, circulating MDSC levels were reduced by half (Tobin et al. 2023).

DHODH inhibitors induce terminal differentiation of acute myeloid leukemia cells, which share a similar progenitor with MDSCs (Christian et al. 2019; Horvat and Lesinski 2022). When tested on a preclinical breast tumor model, a DHODH inhibitor brequinar promoted the maturation of MDSCs and controlled their suppressive effects without decreasing the MDSC level in the tumor (Colligan et al. 2022). Combination with anti‐PD‐1 therapy controlled the tumor growth and decreased the metastasis frequency compared to each therapy (Colligan et al. 2022).

TLRs are transmembrane receptors located in the cell membrane or endosomes of the immune cells, which recognize the pathogen‐associated molecular patterns and induce immune reactions against pathogens (Akira and Takeda 2004). Activating TLRs is beneficial for controlling MDSCs in most cases, but the activation of TLR2 and TLR4 on MDSCs increases MDSC accumulation and upregulates the suppressive functions of the MDSCs (Zhou et al. 2021). On the other hand, TLR3 agonists, such as polyinosinic‐polycytidylic acid (Poly (I:C)), decreased the MDSC level and promoted their maturation to mediate the antitumor response with CAR‐T therapy in preclinical models (Di et al. 2019; Forghani and Waller 2015). TLR9 agonists, CpG oligodeoxynucleotides, also promoted the differentiation of M‐MDSC to macrophages and induced their maturation (Shirota et al. 2012). TLR7/8 agonists such as imiquimod (R837) (Shirota et al. 2012) and resiquimod (R848) (Lee et al. 2014) induced the differentiation of MDSCs into APCs and promoted their T‐cell stimulatory activity. R848 elevated the surface expression of macrophage and dendritic cell markers, MHC markers, and co‐stimulatory molecules, thereby facilitating MDSCs to present antigens to T cells (Spinetti et al. 2016). The R848‐induced differentiation was also observed in human MDSCs (Wang et al. 2015). Phase Ib clinical trial using a TLR8 agonist, motolimod, combined with anti‐epidermal growth factor receptor (anti‐EGFR) induced MDSC differentiation to macrophages ex vivo and reversed the immunosuppressive TME in patients with head and neck squamous cell carcinoma (Shayan et al. 2018), achieving an overall disease control rate of 54% (Chow et al. 2017). Among these TLR agonists, only imiquimod was approved by the Food and Drug Administration (FDA) for topical treatment of superficial basal cell carcinoma (Varshney et al. 2021).

## 4 TLR7 Agonist Delivery for MDSC Control

TLR7 agonists have been used to enhance phagocytosis of APCs, promote dendritic cell maturation, and polarize macrophages toward pro‐inflammatory M1 phenotype for tumor immunotherapy (Rodell et al. 2018; Sheng et al. 2024; Sun, Li, et al. 2022). Upon TLR7 activation, APCs release multiple immunostimulatory cytokines, facilitating CD8^+^ T cell priming and NK cell activation to enhance tumor clearance (Sun, Li, et al. 2022). Importantly, TLR7 agonists reprogram MDSCs through innate immune activation, converting these suppressive cells into pro‐inflammatory APCs.

Despite their therapeutic potential in cancer immunotherapy, TLR7 agonists face significant barriers to clinical translation due to their physicochemical and biopharmaceutical challenges. Oral administration of TLR7 agonists is challenged by low bioavailability due to poor water solubility and short half‐lives (Engel et al. 2011). Side effects also limit the systemic administration of TLR7 agonists. Orally administered TLR7 agonists showed dose‐dependent hematological toxicity, including lymphopenia and anemia, flu‐like symptoms, and vomiting in a clinical trial (Savage et al. 1996). Parenteral administration of TLR7 agonists induced non‐specific immune activation, leading to cytokine storm syndrome and body weight loss in preclinical models (Michaelis et al. 2019; Phuengkham et al. 2019; Sun et al. 2023).

Various drug delivery strategies have been explored to control the release of TLR7 agonists and improve their delivery to APCs or tumor tissues, thereby minimizing systemic exposure and adverse effects. The carriers are designed to enhance solubility, prolong circulation time, and facilitate intracellular trafficking of the drug to endosome—the intracellular site of action. By improving pharmacokinetics and enhancing cellular uptake, the carriers improve the therapeutic efficacy and safety profile of TLR7 agonists, potentiating their use in cancer therapy.

In the following section, we review drug delivery systems developed for R848, a small molecule (314.4 Da) TLR7 agonist, more potent than the FDA‐approved imiquimod and their combinations with chemotherapeutics, photothermal therapy, and immune checkpoint inhibitors. R848 was tested in multiple clinical trials (Table 1); however, the main challenge is its low water solubility (0.3 mg/kg), short terminal‐phase half‐life (~7 h), and cytokine‐induced side effects (Pockros et al. 2007). Consequently, most completed clinical trials have relied on local administration, mainly by topical application. Preclinical and translational efforts have focused on developing local or systemic drug delivery systems to extend the half‐life and prevent nonspecific immune activation, with current clinical trials evaluating intratumoral delivery by a hydrogel formulation and intravenous delivery using a liposomal formulation (Table 1). In this review, we discuss representative approaches for local and systemic delivery of R848, with a specific emphasis on the efforts targeting MDSCs. Other studies focusing on activating innate immune cells, such as DCs and macrophages, or improving overall immune responses are summarized separately in Table 2 (local delivery) and Table 3 (systemic delivery).

**TABLE 1:** Completed and ongoing clinical trials of resiquimod (R848).

Trial number	Phase	Status	Route	Indications	Notes
NCT00470379	Phase I	Completed	Local (topical)	Melanoma	Combined with NY‐ESO‐1b peptide vaccine therapy
NCT00117923	Phase II	Completed	Local (topical)	Common warts	
NCT00117871	Phase II	Completed	Local (topical)	Common warts	
NCT01583816	Phase II	Completed	Local (topical)	Keratosis Lesions	
NCT00116675	Phase II	Completed	Local (topical)	Common warts	
NCT00116662	Phase II	Completed	Local (topical)	Common warts	
NCT00114920	Phase II	Completed	Local (topical)	Common warts	
NCT00175435	Phase I & II	Completed	Local (topical)	Hepatitis B	Combined with Gardasil hepatitis B vaccine
NCT00115141	Phase II	Completed	Local (topical)	Common warts	
NCT01676831	Phase I & II	Completed	Local (topical)	Cutaneous T Cell Lymphoma	
NCT02090374	N/A	Completed	Local (nasal)	Asthma, lung inflammation	
NCT01737580	Phase I & II	Completed	Local (topical)	Influenza	
NCT01808950	Phase I & II	Terminated	Local (topical)	Nodular Basal Cell Carcinoma	Terminated due to safety issue
NCT06021002	N/A	Unknown	Local (nasal)	Asthma, lung inflammation	
NCT00960752	Phase II	Completed	Local (topical)	Melanoma	Combined with gp100 (g209‐2 M) and MAGE‐3 vaccine
NCT00948961	Phase I & II	Completed	Local (topical)	Advanced Malignancies	Combined with CDX‐1401 cancer vaccine and poly:IC
NCT00821652	Phase I	Completed	Local (topical)	Tumors	Combined with NY‐ESO‐1b protein and Montanide adjuvant
NCT02126579	Phase I & II	Completed	Local (topical)	Melanoma	Combined with LPV7 vaccine and tetanus peptide
NCT01748747	Phase I	Completed	Local (topical)	Melanoma	Combined with MART‐1 antigen and Gag:267–274 peptide vaccine
NCT01094496	Phase II	Terminated	Local (topical)	Bladder Cancer	Combined with CDX‐1307 vaccine, GM‐CSF and poly:IC; terminated due to portfolio prioritization and slow enrollment
NCT01204684	Phase II	Completed	Local (topical)	Glioma	Combined with tumor‐cell lysate pulsed‐DCs
NCT06450106	Phase I	Recruiting	Local (intratumoral)	Prostate Cancer	Hydrogel‐based resiquimod formulation
NCT05710848	Phase I & II	Recruiting	Local (intratumoral)	Non‐muscle‐invasive Bladder Cancer	Hydrogel‐based resiquimod formulation
NCT06822998	Phase I	Recruiting	Systemic (intravenous)	Advanced Solid Tumors	Liposomal resiquimod and T‐cell engager formulation

*Note:* ClinicalTrials.gov.

**TABLE 2:** Local R848 delivery for cancer immunotherapy.

Delivery system	Carrier materials	Active ingredients	Target cells	Effects	References
Hydrogel (in situ)	Alginate‐Ca^2+^	R848; iron oxide NP (ferroptosis inducer)	Macrophages	M1 polarization in vitro; 60% survival at day 45 in s.c. 4T1 tumor model compared to 0% in single‐agent‐loaded hydrogel	(Xiao et al. 2022)
Hydrogel (implant)	Polyethylene glycol (PEG) with amine terminal and oxidized dextran (ODEX)	R848; oxaliplatin	—	Combined with anti‐PD‐1, achieved 85% of tumor‐free survival in the peritoneal metastatic colorectal MC38 model, compared to 45% with the free drug combination	(Si et al. 2023)
Hydrogel (implant)	PEG with hydroxylamine terminal and ODEX	Temozolomide; R848; IOX1 (checkpoint inhibitor)	—	90% tumor‐free survival rate in a post‐surgery orthotopic C6 glioblastoma rat model compared to 50% in hydrogel without IOX1; elevated T cell frequency and T cell activation in vivo; activation of antigen presentation pathway in vivo	(Lv et al. 2024)
Hydrogel (in situ)	methoxy poly(ethylene glycol)‐block‐poly(L‐methionine) copolymer (mPEG‐b‐PMet)	R848; doxorubicin; anti‐PD‐1	—	DC and macrophage maturation in vitro; 90% tumor‐free survival rate in the post‐operative B16F10 tumor model compared to 30% in free drug combination; elevated innate and adaptive immune cells the TME and memory T cells in the spleen compared to free drug combination	(Li, Ding, et al. 2024)
Hydrogel (in situ)	poly(D,L‐lactide)‐block‐poly(ethylene glycol)‐block‐poly(D,L‐lactide) (PDLLA‐b‐PEG‐b‐PDLLA)	R848; oxaliplatin	DCs and macrophages	Maturation of DC and M1 polarization in vitro; Extended the median survival to 74.5 days compares to 51 days in free drug combination group in metastatic CT‐26‐Luc tumor model; rejection of tumor rechallenge; increase in central memory T cells in the spleen	(Wang et al. 2023)
Hydrogel (in situ)	polylactic acid (PLA)‐b‐ polyethylene glycol (PEG)‐b‐ poly(N‐isopropylacrylamide) (PNIPAM)	R848; paclitaxel		80% tumor regression in s.c. CT26 colon tumor model compared to 10% in free drug combination	(Vohidov et al. 2020)
Hydrogel (in situ)	PDLLA‐PEG‐PDLLA	R848; CpG; indocyanine green	DCs	Laser induced gelation achieved 80% tumor regression compared to 0% without laser; elevated matured DCs and CD8+ T cells in lymph node	(Jia et al. 2020)
Hydrogel (in situ)	gellan gum	R848; molybdenum polyoxometalate	—	Combination with laser significantly inhibited tumor growth compared to single‐agent‐loaded hydrogel in s.c. 4T1 breast tumor model	(Liu et al. 2022)
Polymeric NPs	PLGA	R848 (LC: 0.13%); indocyanine green	DCs	DC maturation in vitro; Combination with laser decreased tumor burden in s.c. RM9‐Luc prostate cancer model compared to laser alone	(Lin et al. 2021)
Polymeric NPs	PEG‐coated PLGA	R848; poly (I:C); MIP3*α* (chemokine)	DCs	DC maturation in vitro; Combination with tumor vaccine led to 10% and 100% tumor‐free survival in s.c. RMA T cell lymphoma and TC‐1 lung carcinoma model, respectively, compared to 0% in tumor vaccine alone	(Da Silva, Camps, Li, Chan, et al. 2019)
Polymeric NPs	PEG‐coated PLGA	R848; poly (I:C); MIP3*α*; doxorubicin	DCs	DC maturation in vitro; 10% and 50% tumor‐free survival in s.c. TC‐1 lung carcinoma model and MC‐38 colon adenocarcinoma model, respectively, compared to 0% in NPs with doxorubicin only.	(Da Silva, Camps, Li, Zerrillo, et al. 2019)
Polymeric NPs	Chitosan‐coated PLGA	R848; poly (I:C); mesothelin	DCs	DC maturation in vitro; Suppressed tumor growth in orthotopic KPC pancreatic cancer model as preventative or therapeutic treatment	(Ferrari et al. 2025)
Polymeric NPs	Polyethylenimine (PEI)‐conjugated PLGA	R848; monophosphoryl lipid A; CpG	Macrophages	Macrophage uptake in vitro; higher antibody titer in vivo after ovalbumin immunization compared to ovalbumin alone	(Ebrahimian et al. 2017)
Polymeric NPs	Monomethoxy‐PEG‐PDLLA	R848 (LC: 4.98%)	DCs and macrophages	DC maturation and M1 polarization in vitro; Combination with laser and CpG‐loaded Au nanorods suppressed the tumor growth in the B16F10 melanoma model	(Jia et al. 2023)
Polymeric NPs	PEG–PLA, PEG‐(g‐poly((2‐dimethylaminoethyl) methacrylate) (PDMA))‐b‐(2‐(diisopropylamino)ethyl methacrylate) (PDPA)	R848 (conjugated to PEG–PLA); CpG; tumor neoantigen	DCs	DC maturation in vitro; combination with anti‐PD‐1 therapy significantly suppressed tumor growth in s.c. MC38 colon tumor model compared to free drug combination	(Su et al. 2023)
Polymeric NPs	PEI conjugated‐cyclodextrin (𝛽‐CD)	R848; CpG; tumor antigen	DCs	DC maturation in vitro; higher lymph node accumulation compared to free drug; 60% survival at day 50 compared to 0% in free drug combination in s.c. MC38 colon tumor model	(Mao et al. 2023)
Polymeric NPs	PEI conjugated‐deoxycholic acid	R848; ovalbumin (OVA)	DCs	DC maturation in vitro; 60% survival at day 30 compared to 0% in free drug combination group in a s.c. B16‐OVA melanoma model	(Jung et al. 2025)
Polymeric NPs	Hyperbranched amino‐modified 𝛽‐CD	R848 (LC: 3%); siRNA targeting CD47	Macrophages	M1 polarization in vitro; suppressed tumor growth compared to vehicle group in a s.c. CT26 colon tumor model	(Shang et al. 2023)
Polymeric NPs	Thioketal polymer coated with dopamine‐conjugated poly(ethylene‐alt‐maleic acid) (DPEMA)	R848 (LC: 3.8%); doxorubicin; MIP3*α*; anti‐PD‐L1	—	Suppressed tumor growth in a s.c. 4T1 breast tumor model compared to free drug combination	(Banstola et al. 2023)
Polymeric microparticles	Primer‐PEG–PLA; coated with PEG‐grafted cationic polypeptide (PPT‐g‐PEG)	R848; CpG (conjugated to primer‐PEG–PLA); tumor neoantigen	DCs	DC maturation in vitro; Combination of the microparticles and anti‐PD‐1 achieved a 70% survival rate compared to 0% in the free drug combination in s.c. MC38 colorectal tumor model	(Ni et al. 2020)
Lipid‐based NPs	1,2‐Dipalmitoyl‐sn‐glycero‐3‐phosphocholine (DPPC); 1,2‐dis‐ tearoyl‐sn‐glycero‐3‐phosphocholine (DSPC); DSPE‐PEG2K	R848 (LC: 4%–13%)	—	Combined with anti‐PD‐1, led to 60% tumor regression by i.t. injection compared to 20% by intravenous (i.v.) injection combined with ultrasound‐induced hyperthermia in a s.c. neu deletion (NDL) mammary carcinoma tumor model	(Zhang, Tang, et al. 2021)
Lipid‐based NPs	Survivin peptide‐conjugated deoxycholic acid; DPPC; DSPE‐PEG	R848 (LC: 2%–4%); SD‐208 (TGF*β*R1 inhibitor); tumor antigen	DCs	DC maturation in vitro; suppressed tumor growth compared to free drug combination in s.c. B16F10 melanoma model and orthotopic 4T1 breast tumor model	(Kim et al. 2024)
Lipid‐based NPs	Ionizable lipid modified with 𝛽‐cyclodextrin (CD)	R848 (LC: 6.2%); antigen‐encoding mRNA	DCs	DC maturation in vitro; combined with anti‐PD‐L1, led to 80% survival compared to 20% in mice treated with drug‐free NPs in a s.c. MC38 colorectal tumor model	(Qi et al. 2024)
Lipid‐based NPs	1,2‐distearoyl‐sn‐glycero‐phosphoethanolamine (DSPE); dimethyldioctadecylammonium bromide (DDA); immunostimulatory glycolipid trehalose 6,6′ – dibehenate (TDB)	R848 (conjugated to DSPE); H56 tuberculosis antigen	—	High retention at the injection site after i.m. injection with 0.02% lymph node drainage; failed to generate an immune response or antibody production	(Wilkinson et al. 2018)
Lipid‐based NPs	C14‐conjugated G0 dendrimer; ceramide‐PEG	R848 (conjugated to palmitic acid); mRNA encoding OVA antigen	DCs	DC antigen presentation; Suppressed tumor growth compared to NP with negative control mRNA in s.c. EG7‐OVA lymphoma and RM1‐OVA prostate cancer models	(Islam et al. 2021)
Lipid‐based NPs	Amino lipid; 1,2‐dioleoyl‐sn‐glycero‐3‐phosphoethanolamine (DOPE); cholesterol; 1,2‐dimyristoyl‐rac‐glycero‐3‐methoxypolyethylene glycol‐2000 (DMG‐PEG2000)	R848 (conjugated to amino lipid); mRNA (CD40)	DCs	DC maturation in vitro; combined with anti‐CD40, led to 70% survival compared to 25% in NP without mRNA, 0% in the anti‐CD40 only group in a s.c. B16F10 melanoma tumor model	(Yan et al. 2022)

*Note:* LC: Loading capacity = Weight of loaded drug/Weight of NPs.

**TABLE 3:** Systemic R848 delivery for cancer immunotherapy.

Delivery system	Carrier materials	Active ingredients	Target cells	Effects	References
Polymeric NPs	PEG‐PLGA coated with DOTAP	R848; siRNA targeting CD40	DCs	DC maturation; delayed the tumor growth of the orthotopic 4T1 breast tumor model compared to vehicle group	(Li et al. 2023)
Polymeric NPs	PLGA NPs adsorbed to *Escherichia coli*	R848 (LC: 5.9%); Doxorubicin	Macrophages	M1 polarization; suppressed tumor growth of an orthotopic 4T1 breast tumor model	(Wei et al. 2021)
Polymeric NPs	Anti‐PD‐1 conjugated‐PLGA	R848; SD‐208 (TGF*β*R1 inhibitor)	T cells	Extended the survival in the s.c. MC38 colorectal tumor compared to non‐targeted NPs—free anti‐PD‐1 combination	(Schmid et al. 2017)
Polymeric NPs	Poly(2‐oxazoline)	R848 (LC: 15%–41%)	Macrophages	M1 polarization; led to 20% survival compared to 0% in anti‐PD‐1‐treated mice	(Vinod et al. 2020)
Polymeric NPs	PEG; mannose‐modified poly(ethyl methacrylate)‐based polymer	R848 (LC: 7.4%); STING agonist	Macrophages	M1 polarization; suppressed tumor growth in both s.c. and metastatic B16F10 melanoma models compared to vehicle group	(Li, Yi, et al. 2024)
Polymeric NPs	D‐ɑ‐tocopheryl modified with PEG and succinate (TPGS)	R848 (LC: 12.5%); ABT‐737 (chemotherapy)	—	Delayed the tumor growth in s.c. 4T1 breast tumor model compared to vehicle group	(Lang et al. 2024)
Polymeric NPs	PEG or poly(*β*‐amino ester) (PAE) conjugated‐poly(*ε*‐caprolactone) (PCL)	R848 (LC:18%); anti‐CD16; anti‐PD‐L1; anti‐CD16	NK cells	NK cell activation; delayed the tumor growth in s.c. B16F10 melanoma tumor model compared to vehicle group	(Zhang, Fu, et al. 2023)
Polymeric NPs	Boltorn H40 (biodegradable hyperbranched polymer)	R848 (conjugated to carrier, LC: 15%)	DCs	DC maturation; suppressed tumor growth compared to untreated group in a xenograft NDL breast tumor model	(Kakwere et al. 2021)
Polypeptide NPs	Poly(L‐histidine); hyaluronic acid	R848 (LC: 22.5%); Doxorubicin (conjugated to hyaluronic acid)	DCs	DC maturation; suppressed tumor growth in s.c. 4T1 breast tumor model compared to free drug combination	(Liu et al. 2018)
Polypeptide NPs	Octadecanoic acid chain‐modified PD‐1 and PD‐L1 antagonizing peptides via cleavable linker	R848; Doxorubicin	DCs; macrophages	DC maturation; M1 polarization; 80% survival compared to 40% in NPs with non‐cleavable linker at day 40 in a metastatic 4T1 tumor model	(Sun, Yao, et al. 2022)
Polypeptide NPs	Macrophage targeting chimeric peptide with lysine termini	R848 (LC: 3.7%); SHP009 (tyrosine phosphatase 2 inhibitor)	Macrophages	M1 polarization; reduced the metastatic burden compared to free drug in an orthotopic 4T1 tumor model	(Chen, Yan, et al. 2023)
Polypeptide NPs	RGD‐PEG‐b‐ poly‐*γ*‐glutamic acid (PGA)‐g‐triethylene‐tetramine‐bis(dithiocarbamate)‐poly‐L‐histidine	R848 (LC: 18.8%); copper chelator	DCs	DC maturation; controlled the growth of s.c. 4 T1 breast tumor compared to vehicle group	(Zhou et al. 2019)
Polypeptide NPs	*β*‐sheet forming peptide linked to integrin‐targeting peptide	R848 (conjugated to *β*‐sheet forming peptide)	Macrophages	M1 polarization; combined with anti‐PD‐1, led to 100% tumor‐free survival and rejection of tumor rechallenge compared to 0% survival in anti‐PD‐1 only group in a orthotopic 4T1 tumor model	(Zhang et al. 2022)
Polypeptide/polysaccharide NPs	Poly‐L‐histidine; hyaluronic acid	R848; Doxorubicin; cisplatin	DCs; macrophages	DC maturation; M1 polarization; 50% survival rate by 80 days in an orthotopic K7M2 tibiofibular osteosarcoma tumor model compared to 0% in free drug combination	(Zhang, Yuan, et al. 2021)
Polysaccharide NPs	Hyaluronic acid	R848 (conjugated to hyaluronic acid); bexarotene	DCs; macrophages	DC maturation; M1 polarization; suppressed tumor growth in a B16F10‐Luc melanoma model compared to vehicle group	(Sallam et al. 2021)
Polysaccharide NPs	Carboxymethylated alginate	R848 (conjugated to carboxymethylated alginate)	DCs; macrophages	DC maturation; M1 polarization; 50% survival rate by 35 days in a s.c. MFC gastric cancer model compared to 0% with free drug	(Chen, Liu, et al. 2023)
Polysaccharide NPs (nanogels)	𝛽‐cyclodextrin acrylate cross‐linked with 2,2‐dimethacryloyloxy‐1‐ethoxypropane and methacrylamide hyaluronic acid	R848; epigallocatechin‐3‐gallate (EGCG)	DCs	DC maturation; combined with OX40 agonist, led to 40% survival at day 42 compared to 0% in free drug combination in a s.c. B16F10 melanoma model	(Song et al. 2021)
Polysaccharide NPs	Carboxymethyl dextran crosslinked with lysine	R848	Macrophages	M1 polarization; suppressed tumor growth of a s.c. MC38 colorectal tumor model compared to blank NPs	(Sarkar et al. 2024)
Polydopamine NPs	Mesoporous polydopamine NPs coated with cancer cell membrane	R848	DCs	DC maturation; combined with laser and anti‐PD‐L1, led to 100% survival compared to 0% in the NPs‐only group in a s.c. 4T1 tumor model	(Li et al. 2022)
Polydopamine NPs	Mesoporous polydopamine NPs coated with tumor‐ or M2 macrophage targeting peptide anchored red blood cell membrane	R848; metformin	Macrophages	M1 polarization; combined with laser, suppressed tumor growth in a s.c. 4T1 breast tumor model compared to vehicle and laser only	(Huang et al. 2024)
Lipid‐based NPs	DPPC; DSPE‐PEG2000	R848 (LC: 9.7%)	Macrophages	M1 polarization; combined with anti‐EGFR antibody, suppressed tumor growth compared to free drug combination in a s.c. WiDr colorectal adenocarcinoma model	(Li, Somiya, and Kuroda 2021)
Lipid‐based NPs	DPPC; DSPE‐PEG2000	R848; SPII	—	Combined with laser, suppressed tumor growth compared to mice treated with laser and blank NP in a s.c. 4T1 breast tumor model	(Li, Yu, et al. 2021)
Lipid‐based NPs (cationic liposomes)	1,2‐distearoyl‐3‐trimethylammonium‐propane (DSTAP) or R8/A6‐C18; DSPC; cholesterol	R848 (LC: 3.9%–6.6%)	DCs; macrophages	DC increase; combination of oxaliplatin with DSTAP‐R848 or R8‐R848 liposomes led to 60%–70% tumor‐free survival, compared to 30% survival in free drug combination, in a metastatic CT26‐luc colon tumor model	(Chao et al. 2024)
Lipid‐based NPs	Lecithin; cholesterol; mycoplasma membrane	R848; podophyllotoxin	—	Suppressed tumor growth in an orthotopic 4T1‐luc tumor model compared to vehicle control	(Cheng, Yu, et al. 2022)
Inorganic NPs	MSN; PEG	R848 (LC: 10%–17%)	DCs; macrophages	DC maturation; M1 polarization; Suppressed tumor growth in an orthotopic 4T1 breast tumor model compared to vehicle control	(Luo et al. 2025)
Inorganic NPs	MSN; anti‐PD‐L1‐conjuated PEG	R848 (LC: 0.43%); high mobility group nucleosome‐binding protein 1 (HMGN1)	DCs	DC maturation; 100% tumor‐free survival in a s.c. CT26 colon tumor model compared to 0% in free drug combination group	(Huang, Nahar, et al. 2023)
Inorganic NPs	Spiky silica oxide; dimyristoylphosphatidylcholine (DMPC); DSPE‐PEG; cholesterol; apolipoprotein A1; manganese	R848	DCs; macrophages	DC maturation; M1 polarization; 70% survival at day 60 compared to 0% in vehicle group in a metastatic 4T1‐luc breast tumor model	(Tian et al. 2024)
Inorganic NPs	Black phosphorus nanosheets; PLGA	R848	—	Combined with light irradiation, suppressed tumor growth in a s.c. Hepa1‐6 liver cancer model compared to blank NP group	(Liao et al. 2024)

### 4.1 Local Delivery

Local delivery systems are used to enhance drug effect within the TME while minimizing off‐target effects by confining drug distribution to tumor sites and controlling its release. This approach is particularly relevant for treating accessible solid tumors, where direct intratumoral injection is possible (Aznar et al. 2017). Hydrogels and nanoparticles (NPs), which can be intratumorally (i.t.) injected or implanted, are commonly used for local delivery of R848. The main goal is to achieve high intratumoral concentration and limit plasma exposure of the drug, enabling efficient TLR7 activation in the tumor and draining lymph nodes with a minimal risk of systemic cytokine‐related toxicities.

#### 4.1.1 Hydrogel

Hydrogel is a hydrophilic crosslinked polymeric network that can be delivered as pre‐formed implants via surgical insertion, or as injectable solutions that gel in situ. R848 has been co‐delivered with chemotherapy or photothermal agents in hydrogels to generate tumor antigens and activate immune cells in situ simultaneously.

**FIGURE 4:**
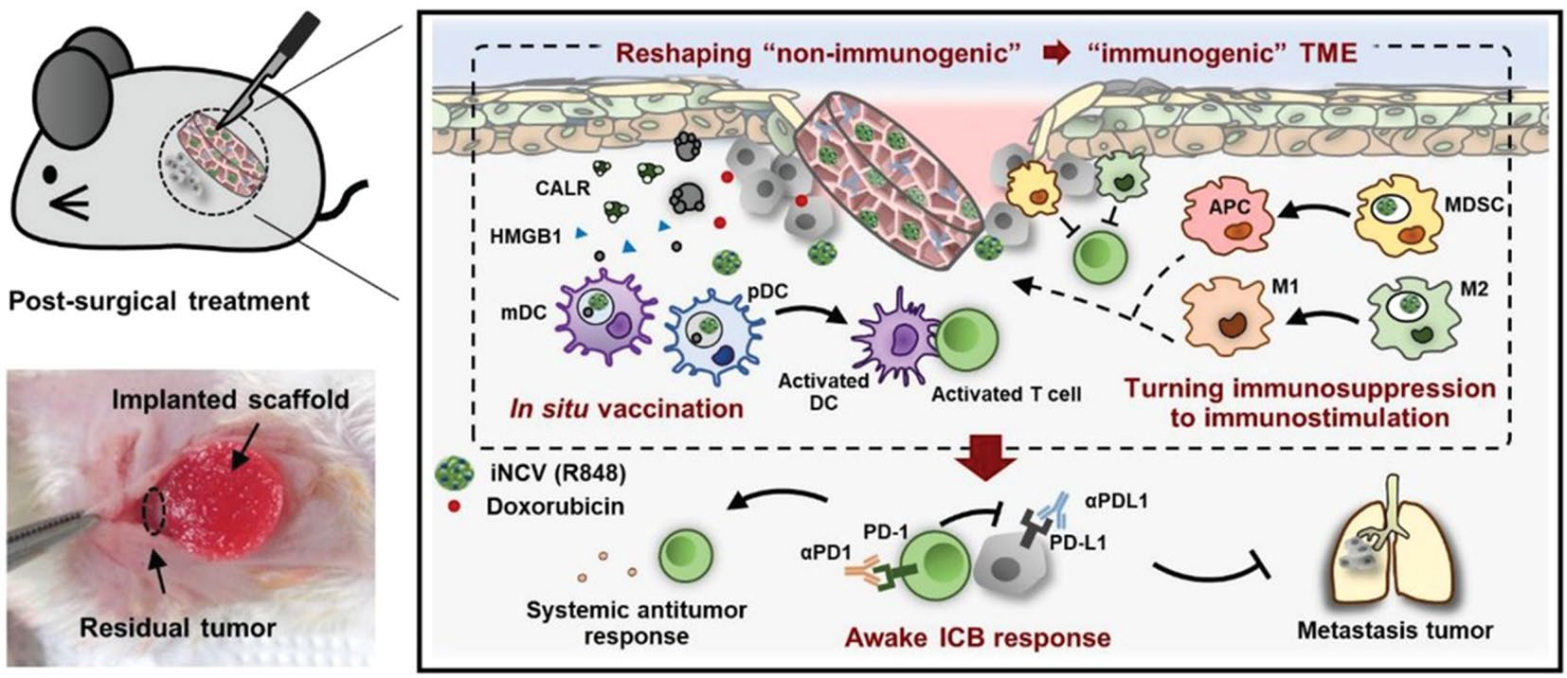
Schematic of hyaluronic acid (HA) scaffold for DOX and R848 PLGA NP (iNCV) delivery and its antitumor mechanism. Reprinted with permission from Phuengkham et al. (2019). Copyright (2019) John Wiley and Sons.

For example, R848 and doxorubicin (DOX, chemotherapy) were co‐delivered to post‐surgical residual tumors by collagen‐hyaluronic acid scaffold, which absorbed interstitial fluid to form a hydrogel depot upon implantation (Figure 4). R848 was first encapsulated in poly(lactic‐co‐glycolic acid) (PLGA) NPs to enhance its cellular uptake to intracellular target—the endo/lysosomal TLR7—of immunosuppressive myeloid cells in TME (Phuengkham et al. 2019). The R848 NPs facilitated macrophage polarization to the M1 phenotype and induced MDSC differentiation into DCs and macrophages in vitro (Phuengkham et al. 2019). Post‐surgical implantation of the scaffold at the tumor resection site inhibited primary tumor growth and metastasis in s.c. 4T1 breast tumor model compared to the free drug combination, and significantly decreased the intratumoral MDSC level, while elevating the T cell frequency and M1/M2 macrophage ratio. The addition of anti‐PD‐L1 or anti‐PD‐1 to the scaffold extended survival from 0% to 50%, compared to the group treated with scaffold only, in post‐operative s.c. 4T1 breast tumor and TC1 cervical cancer models (Phuengkham et al. 2019).

R848 was also combined with photothermal therapy (Revuri et al. 2021). Albumin‐coated manganese dioxide NPs, which function as a photothermal agent and ROS scavenger, were co‐loaded with R848 in Pluronic F127 and hyaluronic acid‐based hydrogel (BAGEL‐R848) (Revuri et al. 2021). BAGEL‐R848 underwent a solution‐to‐gel transition at body temperature, which was further hardened by laser irradiation at 808 nm to sustain the R848 release (Revuri et al. 2021). In a bilateral 4T1 breast tumor model, i.t. injection of BAGEL‐R848, followed by laser ablation, significantly inhibited the tumor growth in both primary and secondary tumors, compared to the group treated with laser and blank hydrogel (Revuri et al. 2021). BAGEL‐R848 and laser ablation also reduced total MDSCs and M‐MDSCs and increased mature DCs in the TME, compared to the groups lacking at least one of the components (Revuri et al. 2021).

#### 4.1.2 Organic (Polymeric, Lipid‐Based) Nanoparticles

R848 has been encapsulated in polymeric NPs to enhance its intracellular delivery or co‐delivery with other drugs. Poly(lactide‐go‐glycolide) (PLGA)‐based NPs were used for co‐delivery of R848 with indocyanine green for photothermal therapy (Lin et al. 2021) or other immunoadjuvants, such as poly(I:C) (Da Silva, Camps, Li, Chan, et al. 2019; Da Silva, Camps, Li, Zerrillo, et al. 2019; Ferrari et al. 2025), monophosphoryl lipid A (MPLA) and CpG (Ebrahimian et al. 2017). The main issue with PLGA NPs for R848 delivery is the low drug loading capacity, defined as the weight of loaded drug divided by the weight of NPs. The reported R848 loading capacity in PLGA NPs ranges from 0.13% (Lin et al. 2021) to 2.8% (Pusch et al. 2021), depending on the preparation method or the polymer type.

Other polymers used for R848 nanoencapsulation include PLA, PDLLA, PEI‐PEG, 𝛽‐CD derivatives, and chitosan derivatives. Monomethoxy‐PEG‐PDLLA micelles encapsulate R848 with a loading capacity of around 5% (Jia et al. 2023). Due to the challenges in drug loading, R848 is often covalently conjugated to the carrier. Transient conjugation (TransCon) R848 was formulated by conjugating R848 to a PEG hydrogel microsphere with a diameter of 70–100 μm, which, upon i.t. injection, extended the plasma half‐life from 10 to 250 h compared to free R848 in rats (Zuniga et al. 2022). The i.t. injected TransCon R848 suppressed the tumor growth in a subcutaneous (s.c.) CT26 colon tumor model compared to the vehicle group and induced an increase in M‐MDSC population in the blood 1 day after treatment (Zuniga et al. 2022).

An amphiphilic copolymer consisting of glycol‐chitosan conjugated with polyaniline, a conductive polymer that serves as a photothermal agent, showed a relatively higher drug loading, around 3.7%–9.4% depending on the feeding ratio of the drug (Chen et al. 2020). In a s.c. CT26 colon tumor model, the combination of intratumoral NP injection and 808 nm laser irradiation achieved a 75% tumor‐free survival rate compared to 0% in mice treated with free R848 or NPs only (Chen et al. 2020). Surviving mice rejected the tumor cell rechallenge, indicating the development of antitumor immune memory (Chen et al. 2020). Importantly, the NP and laser combination induced a significant decrease in intratumoral MDSC levels four days after the treatment (Chen et al. 2020).

R848 has been formulated as liposomes, lipid nanoparticles (LNP), and lipid conjugates with relatively high loading capacity compared to polymeric NPs. Heat‐sensitive 1,2‐dipalmitoyl‐sn‐glycero‐3‐phosphocholine (DPPC)‐based liposomes, which utilized ferrous sulfate as a trapping agent for remote loading, achieved a loading capacity of ~4%–13% (Zhang, Tang, et al. 2021). Similarly, R848 was loaded into a DPPC‐based liposome, along with SD‐208, a TGF‐*β* receptor I kinase inhibitor, and a tumor antigen conjugated with deoxycholic acid, with an R848 loading capacity of 2%–4% (Kim et al. 2024). Modifying a novel ionizable lipid with 𝛽‐CD increased the R848 loading in OVA‐coding mRNA‐loaded LNPs from 4% to 6% compared to the mRNA‐loaded LNPs consisting of commercially used ionizable lipid ALC‐0315, likely due to the increased hydrophobicity (Qi et al. 2024).

#### 4.1.3 Inorganic Nanoparticles

Gold NPs coated with amphiphilic 1‐octanethiol and 11‐mercaptoundecanesulfonic acid were loaded with R848 with 0.3% loading capacity. With a mean diameter of 5 nm, the NPs were drained to the lymph nodes to localized immune activation, which manifested by cytokine expression (Mottas et al. 2019). A mesoporous silica NP (MSN) was functionalized with phenyl groups to enable R848 loading in the pore, with a loading capacity of ~3%, then capped with biotin‐avidin to enable pH‐responsive release of R848, and conjugated with OVA (Wagner et al. 2021). The biotin‐avidin‐capped MSN released R848 at pH 5.5, ensuring endo/lysosomal activation once taken up by the APCs (Wagner et al. 2021). Zr‐based metal–organic frameworks (MOFs), which serve as radiosensitizers, were prepared in different shapes—spherical and flower‐like—and compared for R848 delivery combined with radiotherapy (Zhang et al. 2024). The flower‐like MOFs achieved a higher loading capacity of 13% compared to the 9% in the spherical MOFs due to the higher surface area, and the release of R848 was accelerated in acidic pH (Zhang et al. 2024). The MOFs combined with X‐ray extended the survival of mice, and the efficacy were further improved when combined with anti‐PD‐L1 antibody in a s.c. CT26 colon tumor model (Zhang et al. 2024).

### 4.2 Systemic Delivery

Systemic administration of R848 has been pursued for the treatment of inaccessible or metastatic tumors, where local delivery has limited utility. For systemically delivered drug carriers, it is critical to prevent premature leakage during circulation, facilitate preferential biodistribution to the tumor, and achieve timely drug release at the target tissues. To fulfill these requirements, most drug delivery approaches employ NPs with small sizes (typically < 200 nm) that are amenable to intravenous (i.v.) injection. The NPs are often composed of stimuli‐responsive materials for tissue‐ or cell‐specific drug release and modified with protective surface that promotes prolonged circulation and increased tumor biodistribution via the enhanced permeability and retention (EPR) effect (Matsumura and Maeda 1986). Approaches for systemic delivery of R848 focus on improving dissolution of the drug, which is otherwise poorly soluble in aqueous medium, and promoting drug distribution in metastatic tumor sites and its engagement with APCs or MDSCs.

#### 4.2.1 Polymeric Nanoparticles

Poly(ethylene glycol)‐block‐poly(lactic‐co‐glycolic acid) (PEG‐PLGA) NPs were used to co‐deliver R848 and sorafenib, a tyrosine kinase inhibitor with antiangiogenesis effect (Huang, Xu, et al. 2023). R848 was hydrophobically modified with palmitic acid to increase drug loading in PLGA NPs (5.9%) (Huang, Xu, et al. 2023). PEG was linked to PLGA via a pH‐responsive linker so that it could be detached in the acidity of TME to accelerate the drug release and enhance cellular uptake of the NPs (Huang, Xu, et al. 2023). The NPs extended the circulation half‐life of R848 and increased drug accumulation in the tumor compared to free R848, leading to greater antitumor effect in s.c. and orthotopic Hepa1‐6 liver tumor models (Huang, Xu, et al. 2023).

PLGA NP surface is further decorated with target‐interactive ligands, such as peptide, sugar, or antibody, to enhance the uptake in targeted cell populations (Li, Yang, et al. 2024; Schmid et al. 2017). PEG‐PLGA NPs, loaded with R848 and temozolomide (ICD inducer), were decorated with angiopep‐2 and a mannose analogue. Angiopep‐2 interacts with the low‐density lipoprotein receptor‐related protein 1 (LRP1) of brain endothelial cells, facilitating blood–brain‐barrier transcytosis, whereas the mannose conjugate binds to glucose transporter 1 (GLUT1) of glioblastoma cells and the mannose receptor of macrophages (Li, Yang, et al. 2024). Therefore, the modified NPs showed a higher accumulation in the brain and a higher glioma‐to‐brain ratio compared to the unmodified PLGA NPs, leading to 50% survival in an orthotopic glioblastoma tumor model compared to 0% in free drug combination or unmodified NPs (Li, Yang, et al. 2024).

Alternatively, cell membrane is used to functionalize NP surface to exploit native cell–cell interactions. R848 was loaded in polyethyleneimine/albumin‐crosslinked NPs, which were coated with a hybrid of platelet and neutrophil membrane containing integrin and CXCR4, further modified with IR820 (Sheng et al. 2024). The cell membrane‐coated NPs exhibited higher tumor accumulation compared to the uncoated NPs and were primarily taken up by macrophages within the tumor (Sheng et al. 2024). A combination of the membrane‐coated NPs with laser irradiation and anti‐CD47 antibody suppressed tumor growth compared to the membrane‐coated NPs alone in a post‐surgery 4T1 breast tumor model. The membrane‐coated NPs combined with laser irradiation induced a significant decrease in the MDSC frequency in the tumor compared to the PBS group, which was partly attributed to the cell membrane absorption and neutralization of the tumor‐associated factors and chemokines responsible for MDSC recruitment and expansion (Sheng et al. 2024).

**FIGURE 5:**
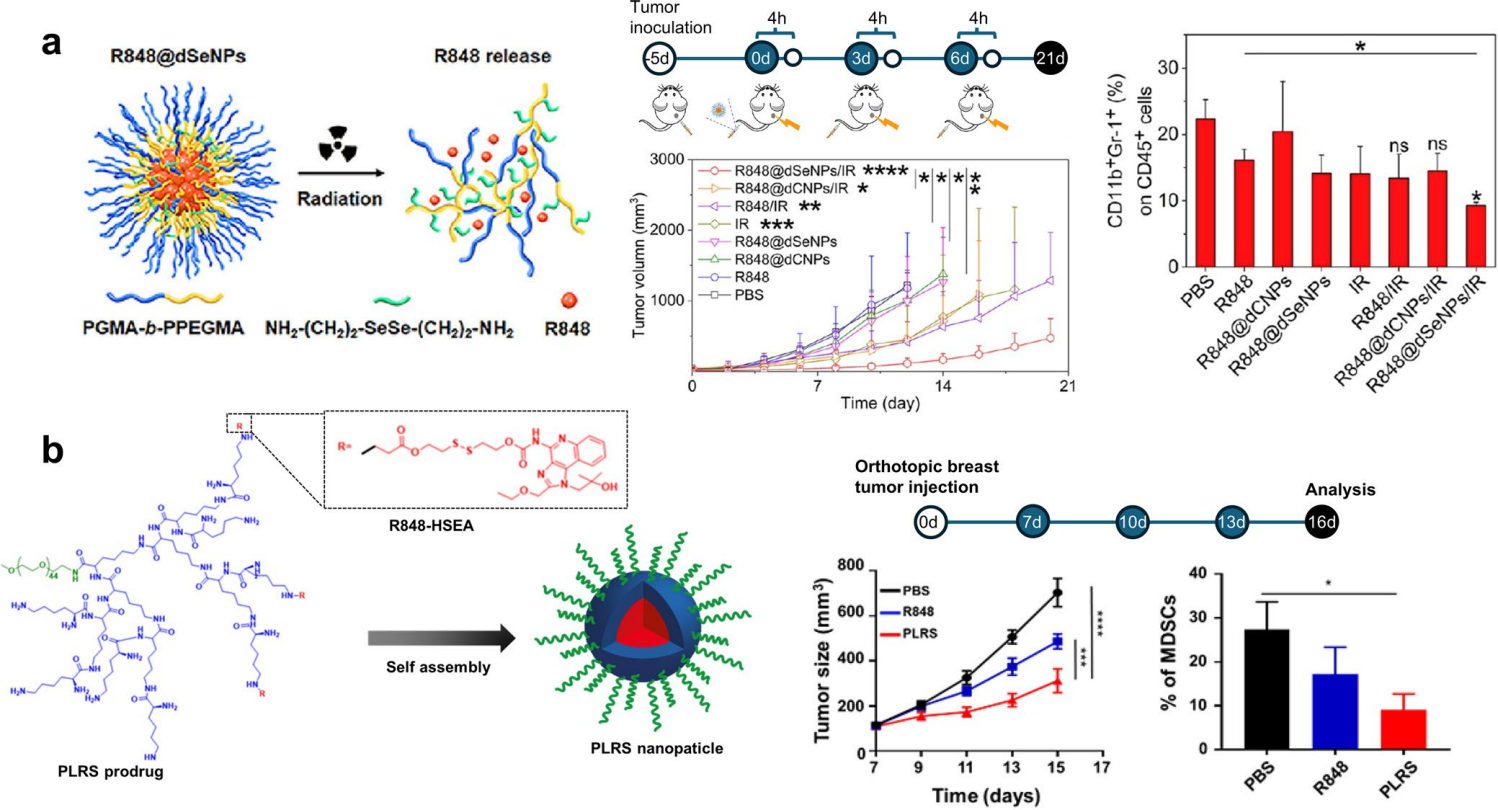
(a) Schematic of R848 in diselenediamine‐crosslinked PGMA‐b‐PPEGMA NPs (R848@dSeNPs); antitumor effect of R848@dSeNPs after i.v. injection in combination with X‐ray irradiation (IR, 4 Gy, 4 h post NP injection); MDSC frequency in tumor 14 days after the first i.v. administration in an s.c. 4T1 breast tumor model (Wu et al. 2024). Copyright (2024) American Chemical Society. (b) Schematics of PLRS, disulfide‐containing acrylate prodrug of R848 (R848‐HSEA) linked to PEG and lysine dendrimer (PL), and its self‐assembly into PLRS NP; antitumor effect and MDSC frequency (on 3 days post the last injection) after i.v. administration in an orthotopic 4T1 breast tumor model. Mice received three doses of PBS, unformulated free R848, or PLRS NPs at 2 mg/kg R848 equivalent (Li, Shu, et al. 2024). Copyright (2024) American Chemical Society.

R848 has been delivered with an amphiphilic block copolymer poly(glycidyl methacrylate)‐block‐poly(methacrylic acid ester of polyethylene glycol) (PGMA‐b‐PPEGMA) NPs, crosslinked via diselenide linkers, which degrade upon X‐ray irradiation (Wu et al. 2024) (Figure 5a). The NPs barely released any loaded R848 without X‐ray irradiation but slowly released R848 over 5 days after irradiation. The NPs combined with irradiation suppressed the tumor growth in a s.c. 4T1 breast tumor model and decreased the MDSC frequency in the tumor‐draining lymph nodes compared to free R848 (Wu et al. 2024).

R848 was conjugated to the disulfide‐containing acrylate and linked to PEG and lysine dendrimer (PLRS), which self‐assembled into NPs that released R848 under a reductive environment (Li, Shu, et al. 2024) (Figure 5b). PLRS NPs led to significant decreases in MDSC levels in the tumor on 3 days post the last treatment (Li, Shu, et al. 2024). PLRS NPs or their combination with anti‐PD‐1 controlled the growth of the orthotopic 4T1 breast tumor model, compared to anti‐PD‐1 alone (Li, Shu, et al. 2024).

#### 4.2.2 Polysaccharide NPs

A R848‐loaded 𝛽‐cyclodextrin (CD) NP was prepared by crosslinking succinyl‐𝛽‐CD with lysine (Rodell et al. 2018). The NPs had an average size around 30 nm and drug loading of 10.4% (Rodell et al. 2018). The CDNPs were distributed preferentially in the tumor at 24 h post i.v. injection and primarily taken up by macrophages within the tumor (Rodell et al. 2018). These CDNPs crossed the blood–brain barrier (BBB) and accumulated in the glioma, where they were mostly taken up by myeloid cells but not in the healthy brain (Turco et al. 2023). In a mouse model of orthotopic Gl261 glioma, CDNPs suppressed the tumor growth and achieved 5% complete response compared to 0% in free R848 (Turco et al. 2023). M‐MDSCs decreased in blood, and CD4^+^ and CD8^+^ T cells increased in the tumor of the CDNP‐treated group, compared to the blank NP group (Turco et al. 2023). T cell or NK cell depletion did not affect the anti‐tumor efficacy of the CDNPs, suggesting myeloid cells as the major effector cells in controlling glioma growth (Turco et al. 2023). While the CDNPs were very potent, slight systemic toxicity, such as body weight decrease, was observed due to the rapid release of R848 from the carrier (Turco et al. 2023). To prevent adverse reactions, R848 was modified with adamantane before loading into CDNPs (Rodell et al. 2019).

#### 4.2.3 Polydopamine Nanoparticles

Polydopamine (PDA) NPs are formed by oxidizing and polymerizing dopamine in alkaline conditions. PDA NPs can be used as a carrier of a photothermal agent and R848, where the cargos are loaded via *π*–*π* stacking and hydrogen bonding (Lu et al. 2019). R848 and carbon dot, a photothermal agent, were loaded onto the surface of PEG‐coated PDA NPs, with R848 loading capacity of 1%–4% (Lu et al. 2019). Acidic pH and laser irradiation accelerated the release of R848 from the NPs (Lu et al. 2019). High tumor and liver accumulation was observed after systemic administration. Combination with laser irradiation significantly controlled the tumor growth of a s.c. 4T1 breast tumor model (Lu et al. 2019).

#### 4.2.4 Lipid‐Based Nanoparticles

R848 has been delivered with 1,2‐distearoyl‐sn‐glycero‐3‐phosphocholine (DSPC) liposomes, which co‐encapsulated indocyanine green (sonosensitizer) and perfluoropropane (C3F8), a gas that facilitates ultrasound‐induced liposome disruption (Chen, Wu, et al. 2023). The liposome only suppressed tumor growth when combined with ultrasound in a s.c. Hepa1‐6 mouse liver tumor model (Chen, Wu, et al. 2023). The TME showed a 50% decrease in MDSC frequency in the liposome‐ultrasound combination group and a 25% decrease in the free R848 group (Chen, Wu, et al. 2023).

**FIGURE 6:**
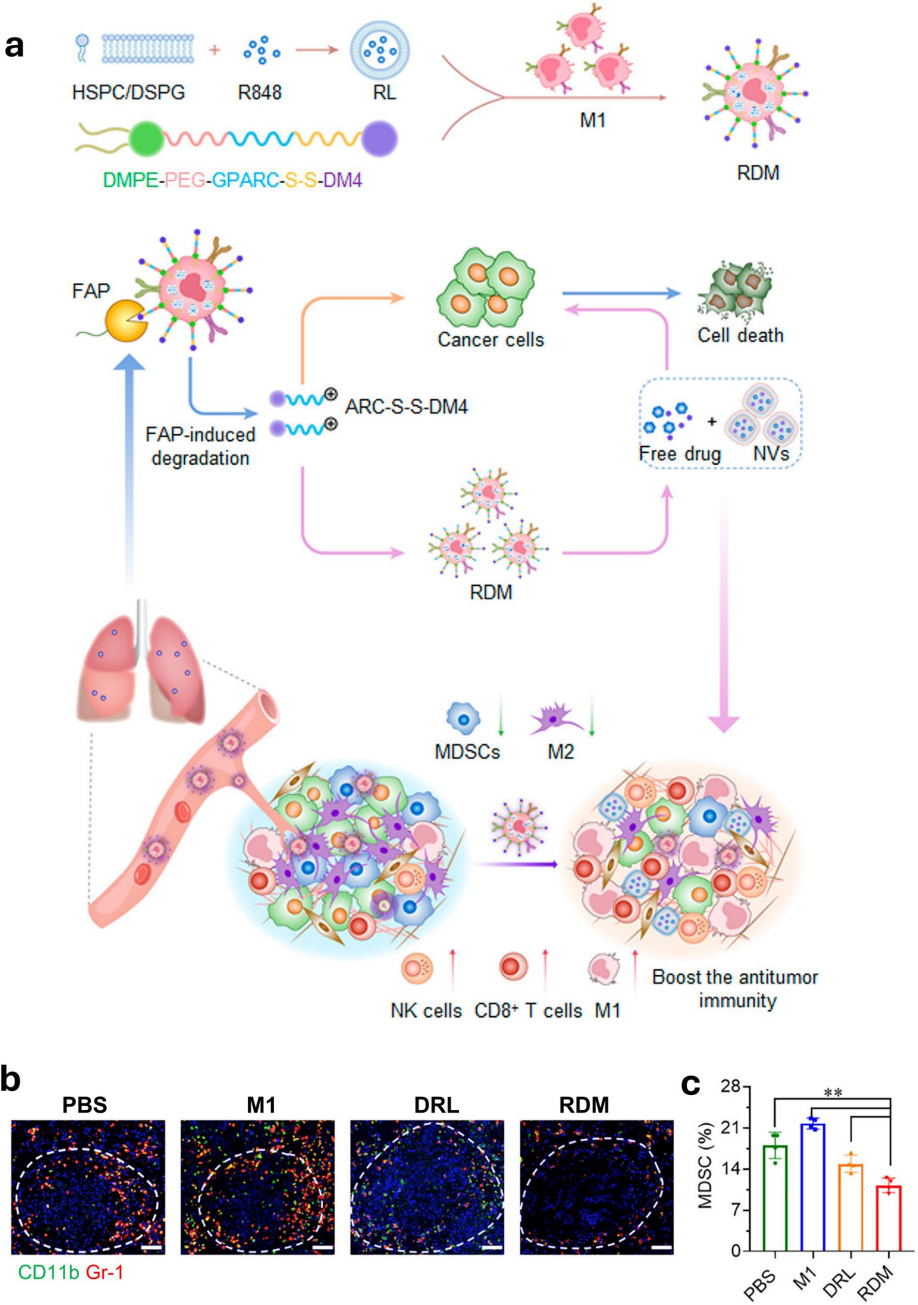
(a) Schematic of R848/DM4‐loaded macrophage (RDM) preparation and the mechanism of modulating TME. R848 liposomes (RL) were loaded in M1 macrophages modified with phospholipid‐conjugated DM4. (b) Lung metastatic lesion (blue, DAPI; green, CD11b; red, Gr‐1) and (c) MDSC frequency in the lung by flow cytometry in a metastatic 4T1 breast tumor model treated with PBS, M1 macrophage, R848‐loaded liposomes (DRL), and RDM at 52 μg/kg R848 equivalent. Reprinted with permission from Xu et al. (2023). Copyright (2023) American Chemical Society.

R848‐encapsulated liposomes were loaded in RAW264.7‐derived M1 macrophages, with phospholipid‐conjugated DM4, a chemotherapeutic agent, incorporated in the cell membrane (Figure 6a) (Xu et al. 2023). Intravenously administered, the R848/DM4‐loaded macrophages (RDM) infiltrated the lung, delivering R848 and DM4 to the metastatic lesions (Xu et al. 2023). RDM significantly decreased the frequency of MDSCs in the 4T1 metastatic lung compared to the PBS control group (Figure 6b,c), and extended the survival curve in the 4T1, EMT6, and Renca lung metastatic tumor models (Xu et al. 2023).

To facilitate the loading in lipid‐based NPs, R848 is conjugated to lipid prior to NP loading. Cholesterol‐conjugated R848 was incorporated in a 1,2‐dioleoyl‐sn‐glycero‐3‐phosphate (DOPA)‐ and 1,2‐dioleoyl‐sn‐glycero‐3‐phosphocholine (DOPC)‐based NPs together with Ce6 photosensitizer (Jiang et al. 2023). The NPs extended the half‐life of R848 compared to free R848 and increased the accumulation of Ce6 in the tumor after systemic administration. The combination of NPs with light irradiation suppressed the tumor growth in s.c. MC38 colorectal tumor and orthotopic 4T1 tumor, showing elevated DC, macrophage, and MDSC levels in the MC38 tumor compared to PBS or free combination group (Jiang et al. 2023).

#### 4.2.5 Inorganic Nanoparticles

Inorganic materials, such as silica, iron, and manganese, were used as R848 carriers (Huang, Nahar, et al. 2023; Tian et al. 2024; Wang et al. 2025). Iron and tetrakis (4‐carboxyphenyl) porphyrin (TCPP) metal–organic frameworks (Fe‐MOFs) were loaded with R848 and stabilized with PEG. In the reductive intracellular environment, the Fe‐TCPP‐R848‐PEG NP released Fe^2+^ for ferroptosis, TCPP for photothermal effect, and R848 for APC activation (Fan et al. 2024). Combination of Fe‐TCPP‐R848‐PEG NP with laser extended the survival time and decreased the metastatic burden in a s.c. B16F10 melanoma tumor model (Fan et al. 2024).

#### 4.2.6 Cell‐ or Bacteria‐Derived Carriers

**FIGURE 7:**
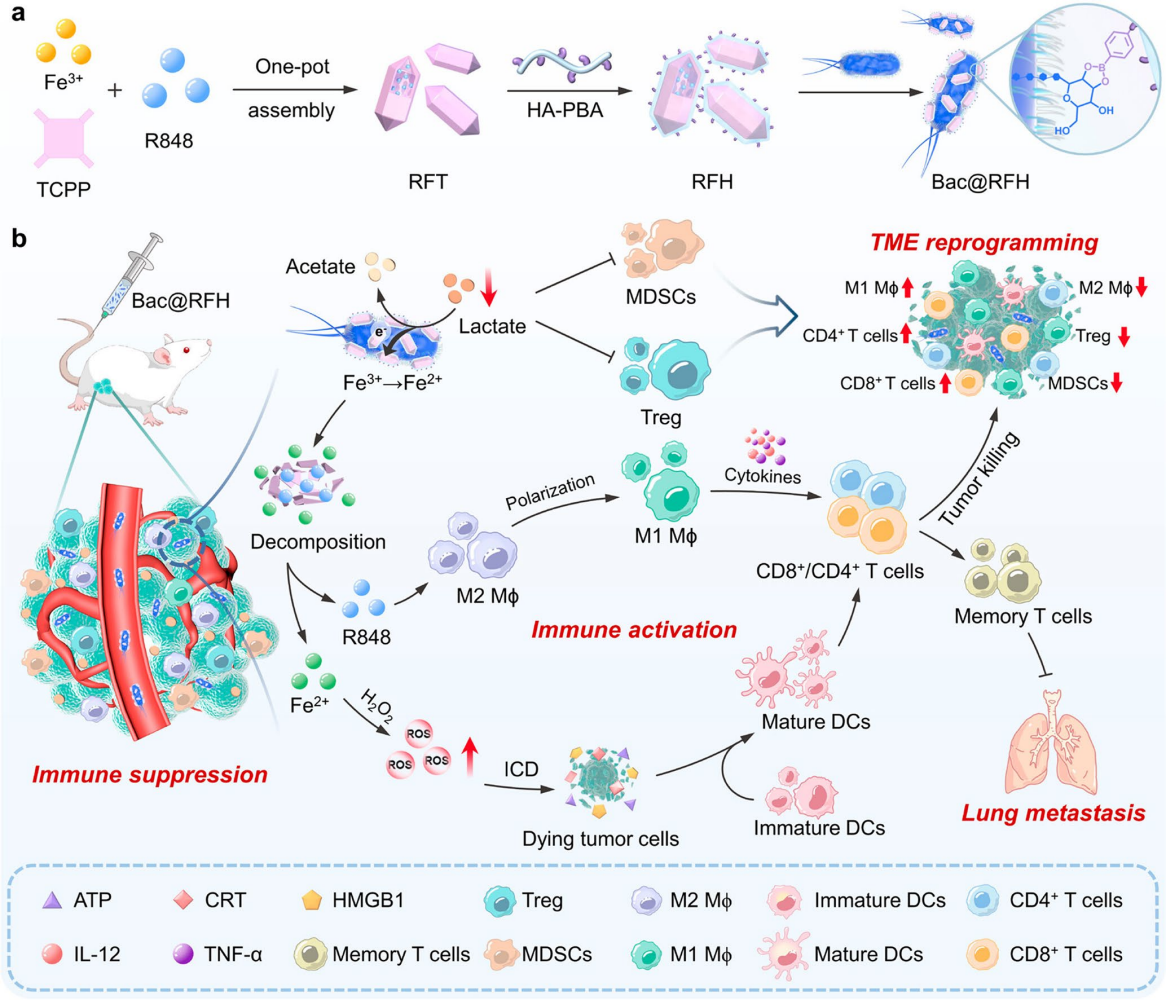
(a) Schematic of Bac@RFH preparation. R848 was loaded in hyaluronic acid‐coated Fe‐TCPP metal–organic framework (RFH), which was covalently linked to *Shewanella oneidensis* (Bac@RFH). (b) Mechanism of Bac@RFH in reprogramming TME. Reprinted with permission from Zhang, Jin, et al. (2023). Copyright (2023) American Chemical Society.

Cell membrane‐derived vesicles, cells, or bacteria have recently been pursued as drug delivery systems, with the main advantages being biocompatibility and tumor tropism (Lin et al. 2024). Engineered macrophage cellular vesicles (EMCVs) utilized M1 macrophage membranes labeled with indocyanine green for the delivery of R848 (Lin et al. 2024). Following i.v. administration, the EMCVs first accumulated in the liver and persisted for 1 day. The accumulation in the tumor gradually increased and reached its highest level at 36 h, suggesting potential redistribution of the EMCVs (Lin et al. 2024). The combination of EMCVs and laser suppressed the tumor growth in a s.c. 4T1 breast tumor model compared to the untreated control group (Lin et al. 2024). In another drug delivery system, the outer membrane vesicles secreted by *E. coli*, fused with ceramide, were loaded with R848 and INCB024360 (IDO inhibitor) with a R848 loading capacity of 6% (RILO). Bone marrow‐derived macrophages were coated with tumor‐targeting peptides and fed with the RILO (RILO@MG), which induced in situ secretion of R848‐containing exosomes once they reached the tumor site. Compared to cell‐free RILO, the RILO@MG exhibited higher tumor accumulation and enhanced anti‐tumor efficacy in s.c. and orthotopic H22 hepatocellular carcinoma tumor models (Liu et al. 2024). Similarly, lactate‐consuming bacterium, *Shewanella oneidensis*, was covalently linked to Fe‐metal–organic framework, which was loaded with R848 (with a R848 loading capacity of 12.7%) and coated with hyaluronic acid (RFH) (Figure 7a) (Zhang, Jin, et al. 2023). The RFH‐bound bacteria (Bac@RFH) metabolized lactate in the TME and reduced Fe^3+^ to Fe^2+^, decreasing the lactate concentration to prevent recruitment of immunosuppressive cells and inducing ferroptosis of tumor cells (Figure 7b) (Zhang, Jin, et al. 2023). Upon systemic administration, Bac@RFH accumulated in the tumor better than RFH due to the bacterial tropism toward the hypoxic TME (Zhang, Jin, et al. 2023). Bac@RFH led to 70% survival by itself and 100% survival by 75 days when combined with anti‐PD‐1 in the s.c. CT26 colon tumor model. It achieved a significant reduction in MDSC frequency in the TME compared to the untreated control (Zhang, Jin, et al. 2023).

## 5 Conclusion and Future Perspective

MDSCs, abundant in TME and further amplified with cancer therapies, exert diverse immunosuppressive functions. Therefore, MDSCs have emerged as critical targets in cancer immunotherapy. However, reducing the MDSC population and blocking their functions alone may be insufficient to control aggressive tumor progression. To enhance therapeutic outcomes, MDSC‐regulating agents are often combined with conventional therapies, such as chemotherapy, photothermal therapy, and immune checkpoint blockade, in preclinical tumor models and early clinical trials, showing positive outcomes (Chen et al. 2020; Tobin et al. 2023; Weiss et al. 2021; Zhang, Jin, et al. 2023). Nonetheless, additional clinical evidence is needed to validate the impact of MDSC downregulation on tumor control and to determine whether MDSC‐regulating strategies are broadly applicable.

TLR7 agonists control MDSCs by downregulating their immunosuppressive functions and reprogramming them into pro‐inflammatory APCs. Additionally, TLR7 agonists like R848 stimulate innate and adaptive immune cells, including DCs, macrophages, and T cells in the TME (Sun, Li, et al. 2022). Due to these comprehensive immunostimulatory effects, R848 is expected to outperform other MDSC‐controlling strategies. Currently, TransCon R848, an R848 conjugated to a PEG hydrogel microsphere, is under clinical investigation as an intratumoral therapy for patients with advanced solid tumors (Zuniga et al. 2022). On the other hand, several TLR7 agonist prodrugs evaluated in Phase 1 or Phase 1/2 clinical trials for intratumoral or systemic administration were terminated, likely due to severe adverse reactions associated with systemic TLR7 activation (Mahalingam et al. 2024; Siu et al. 2020; Wang et al. 2020). Therapeutic use of TLR7 agonists is further complicated by their poor water solubility and unfavorable biopharmaceutical properties.

The significance of MDSC control, the therapeutic potential and side effects of TLR7 agonists, and associated biopharmaceutical challenges present unique opportunities for pharmaceutical scientists. As summarized in this review, which centers on R848 formulations, extensive efforts have been made to improve drug dissolution, control release kinetics, enhance tumor accumulation, and enable co‐delivery with other therapies. NP formulations, leveraging prior knowledge and technical advances to achieve tumor‐targeted drug delivery, have been at the forefront of these strategies. A major challenge, however, is the low drug loading capacity, ranging from 0.1% to 7% in conventional NPs, such as PLGA NPs or liposomes (Chao et al. 2024; Lin et al. 2021; Wei et al. 2021). The reliance on high carrier content introduces uncertainties regarding immunological and toxicological consequences of the products. This limitation has driven the modification of drug molecules and/or overengineering of carriers, increasing complexity and regulatory hurdles for clinical translation.

Notably, the potential of TLR7 agonists to reprogram immunosuppressive MDSC populations remains underexplored. Most studies emphasize their roles in attracting and activating APCs such as DCs and macrophages, rather than controlling MDSC dynamics implicated in tumor growth. Consequently, delivery strategies have primarily focused on concentrating drugs within tumors by local or systemic administration. However, given that secondary lymphoid organs serve as main reservoirs for MDSCs, targeting these sites—in addition to tumors—could be advantageous. Such an approach should also consider the optimal timing of intervention based on MDSC progression throughout disease development and associated therapies, which would require personalized diagnosis based on individual immune profiling. In this regard, it is worth considering the clinically established correlation between MDSC level and disease status (Bergenfelz et al. 2015; Diaz‐Montero et al. 2009; Hao et al. 2021; Sun et al. 2012). Numerous clinical studies use circulating MDSC levels for determining cancer stage, metastatic tumor burden, and therapeutic response to chemotherapy (Bergenfelz et al. 2015; Diaz‐Montero et al. 2009; Kotwal et al. 2023; Markowitz et al. 2015; Sade‐Feldman et al. 2016; Sun et al. 2012; Wesolowski et al. 2017). These studies indicate that the circulating MDSC level is not only a therapeutic target but also a clinically measurable indicator of disease progression, which may be used for designing a personalized regimen for R848 administration.

## Funding

This work was supported by NIH R01 CA232419 (YY) and NIH R01 CA258737 (YY). The authors also acknowledge the support from the Purdue Institute for Cancer Research, NIH grant P30 CA023168.

## Ethics Statement

This article does not contain any studies with human participants or animals performed by any of the authors.

## Conflicts of Interest

The authors declare no conflicts of interest.

## Data Availability

Data sharing not applicable to this article as no datasets were generated or analysed during the current study.
